# Blood lipid metabolism and the risk of gallstone disease: a multi-center study and meta-analysis

**DOI:** 10.1186/s12944-022-01635-9

**Published:** 2022-03-02

**Authors:** Min Zhang, Min Mao, Chi Zhang, Fulan Hu, Ping Cui, Guangcan Li, Jia Shi, Xin Wang, Xuefeng Shan

**Affiliations:** 1 Department of Epidemiology, School of Public Health and Management, Chongqing Medical University, Chongqing, 400016 China; 2 Department of Pathology and Southwest Cancer Center, First Affiliated Hospital of Army Medical University, Chongqing, 400016 China; 3 Department of Prevention, Tianjin Medical University Cancer Institute and Hospital, Tianjin, 300060 China; 4 Department of Epidemiology and Health Statistics, School of Public Health, Shenzhen University Health Science Center, Shenzhen, 518061 Guangdong China; 5 Department of Public Health, Jining Medical University, Jining, 272067 China; 6 Department of Pharmacy, The People’s Hospital of Kaizhou District, Chongqing, 405400 China; 7 Department of Clinical Laboratory, the Second Affiliated Hospital of Dalian Medical University, Dalian, 116027 Liaoning China; 8 Department of Epidemiology and Biostatistics, Sichuan University West China School of Public Health and West China Fourth Hospital, South Renmin Road, Wuhou District, Chengdu, 610041 China; 9 Department of Pharmacy, The First Affiliated Hospital of Chongqing Medical University, No.1 Road Youyi Road, Yuanjiagang Community, Yuzhong District, Chongqing, 400016 China

**Keywords:** Gallstone disease, Lipids, Dyslipidemias, Cholesterol, Triglycerides

## Abstract

**Background:**

Gallstone disease (GSD) is a common and costly biliary disorder. Multiple studies have investigated the associations between blood lipid metabolism and GSD risk; however, the results are inconsistent. This research aimed to comprehensively evaluate the relationships among serum total cholesterol, low-density lipoprotein (LDL) cholesterol, high-density lipoprotein (HDL) cholesterol, triglycerides and GSD risk.

**Methods:**

Firstly, a multi-center cross-sectional study was carried out. Subjects who participated in the health examination in three hospitals between January 2015 and May 2020 were recruited. Multivariable logistic regression was used to investigate blood lipid metabolism associated with GSD risk. Then, a meta-analysis was performed to verify the associations further. Medline and Embase databases were systematically searched before June 10, 2021. The DerSimonian and Laird random-effect model was utilized when the heterogeneity was high; otherwise, fixed-effect model was adopted.

**Results:**

There were 548,934 eligible participants included in the multi-center study, and 45,392 of them were diagnosed with GSD. The results demonstrated that total cholesterol and HDL cholesterol were negatively associated with GSD risk in both high vs. low model and per mmol/L increase model, while triglyceride was positively associated with GSD risk in the per unit increase model. In the meta-analysis, 104 studies with approximately 3 million participants were finally included. The results verified that HDL cholesterol [odds ratio (OR) = 0.636, *P* = 5.97 × 10^− 16^ in high vs low model; OR = 0.974, *P* = 6.07 × 10^− 05^ in per unit model] and triglyceride (OR = 1.192, *P* = 3.47 × 10^− 05^ in high vs. low model; OR = 1.011, *P* = 5.12 × 10^− 05^ in per unit model) were related to GSD risk in the two models.

**Conclusions:**

The findings indicated that low HDL cholesterol levels and high triglyceride levels were risk factors for GSD. This study provides a basis for identifying the population at high risk for GSD and implementing tertiary prevention strategies for GSD, thus contributing to GSD prevention as well as disease burden relief.

**Supplementary Information:**

The online version contains supplementary material available at 10.1186/s12944-022-01635-9.

## Background

Gallstone disease (GSD) is one of the most common biliary diseases, afflicting about 10–20% of the population in European countries [1] and 5–8% in Asian countries [2]. Most GSD patients are asymptomatic, and approximately 20% of them develop abdominal pain and other biliary complications during their lifetime, which require surgical treatment [3]. In the United States, over 700,000 cholecystectomies are performed annually, costing more than 6 billion dollars [1]. Besides, gallstone is a high-risk factor for a wide array of diseases, including biliary tract cancer [4], colorectal cancer [5], cardiovascular diseases [6], and even mortality [7], posing substantial healthcare and economic burden on nations. Therefore, clarifying the pathogenesis and risk factors for GSD is essential to provide prophylactic strategies for the prevention of GSD.

Most gallstones are composed of cholesterol, and hence the role of cholesterol metabolism in the mechanism of gallstone formation has long been a research focus in the etiology of GSD. Several studies have evaluated the relationship between serum lipids levels and GSD risk [2, 8]. However, there is a considerable discrepancy among the findings due to the differences in the study design, sample size, subjects’ ethnicity and definition of dyslipidemia, thus limiting the strength and application of these pieces of evidence.

In this research, a multi-center cross-sectional study in China was firstly conducted to estimate the associations between serum lipids [e.g., total cholesterol (TC), high-density lipoprotein (HDL) cholesterol, low-density lipoprotein (LDL) cholesterol and triglyceride] and GSD risk. Additionally, meta-analysis was conducted to further verify these associations.

## Methods

This cross-sectional study was performed in compliance with the guidelines of the Strengthening the Reporting of Observational Studies in Epidemiology (STROBE) statement [9].

### Study population

Participants in this multi-center cross-sectional study were recruited from the physical examination centers between January 2015 and May 2020 in three hospitals in China. The three hospitals are the First Affiliated Hospital of Chongqing Medical University Jinshan Hospital, the People’s Hospital of Kaizhou District in Chongqing city, and Tianjin Medical University Cancer Institute and Hospital. The participants who met the following criteria were recruited: (i) underwent ultrasonography examination; (ii) had complete demographic, anthropometric and biochemical indexes, including age, gender, height, weight, waist circumference, blood glucose, diastolic/systolic blood pressure (DBP/SBP), and indicators for liver function and kidney function. If the subject participated in multiple health examinations, the latest data were selected. This research was approved by the ethics committee of West China Fourth Hospital and West China School of Public Health, Sichuan University (Gwll2021055), and was conducted according to the ethical guidelines of the 1964 Declaration of Helsinki and its later amendments.

### Laboratory examinations

The fasting blood samples were collected from the subjects, and then measured in laboratories within one hour. The biochemical analyzers were utilized to estimate the serum concentrations of HDL cholesterol, LDL cholesterol, TC, triglyceride, fasting blood glucose (FBG), total bilirubin (T-bil), alanine transaminase (ALT), aspartate transaminase (AST), creatinine (Cr), uric acid (UA), and urea nitrogen (UN). All biochemical indexes were measured by following the international standard protocol independently in each hospital.

### Definitions

Ultrasonography was performed by experienced radiologists. The diagnosis of GSD was based on one of the following two criteria or both: (i) one or more hyperechoic structures in the gallbladder or biliary system, which were acoustic shadowing or gravity-dependent; (ii) no sight of the gallbladder in patients who underwent cholecystectomy due to gallstones. In this cross-sectional study, the lipid markers were classified into three groups according to the Chinese adult dyslipidemia prevention guide (2017 edition), TC (mmol/L): > 5.7, 3.1–5.7, and < 3.1; triglycerides (mmol/L): > 1.7, 0.4–1.7, and < 0.4; LDL cholesterol (mmol/L): > 3.1, 2.07–3.1, and < 2.07; and HDL cholesterol (mmol/L): > 2.0, 0.9–2.0, and < 0.9.

### Meta-analysis

The current meta-analysis was conducted according to the Preferred Reporting Items for Systematic Reviews and Meta-Analyses (PRISMA) statement [10] and the Meta-Analysis of Observational Studies in Epidemiology (MOOSE) guidelines [11]. This meta-analysis has been registered to the International Prospective Register of Systematic Reviews (PROSPERO; registration ID: CRD42020218747).

In this meta-analysis, two corresponding authors (X.S. and X.W.) developed the search strategy, inclusion criteria, and exclusion criteria. Two authors (M.Z. and M.M.) independently conducted literature searching, article selection, data extraction, and quality evaluation. All inconsistent data were discussed and resolved by the corresponding authors. The PubMed and Embase databases were searched to screen the relevant researches published in English before June 10, 2021. The exact keywords are listed in Additional file 1.

Publications were eligible to be included if: (i) they were observational studies conducted in humans, including cross-sectional, case-control, and cohort studies; (ii) the primary outcomes were risk of GSD and/or mean difference in lipid levels between GSD patients and healthy individuals; (iii) they directly provided risk estimates such as hazard ratio (HR), relative risk (RR) and odds ratio (OR) with 95% confidence intervals (CIs), coefficient β and standard error (SE), or provided enough data to calculate these risk estimates; or (iv) they provided mean and standard deviation (SD) in both cases and controls. When the same population was used in multiple research papers, the most recent study or the one with the largest sample size were selected. Irrelevant studies and relevant literature reviews with insufficient data were excluded.

Data were extracted from a predesigned sheet containing the PubMed Unique Identifier (PMID), first author, publishing year, exposure factor, source of population, geographic background, study period, study design, sample size of cases and controls, matched information, number of male and female participants, effects of associations and the corresponding 95%CIs, coefficient β, SE or *P*-value (for studies using multiple adjusted models, the most fully adjusted estimates were extracted), and mean levels of lipids and SD in GSD and non-GSD groups.

Risk of bias and quality assessment were conducted for the included cohort and case-control studies by the Newcastle-Ottawa scale (NOS), and the Agency for Healthcare Research and Quality (AHRQ) for cross-sectional studies as suggested [12, 13]. NOS estimates study quality based on three criteria (the comparability of the groups; selection of the study groups; and ascertainment of outcomes of interest) with a full score of nine. It is assigned as high quality if a study obtains more than six scores, moderate quality if obtains 46 scores, and low quality if obtains less than four scores. AHRQ estimates study quality based on 11 items with a total of 11 scores. Each item answered with “yes” (one score), “no” or “not reported” (zero score). A study is assigned as high, moderate or low quality when it gets eight-11, four-seven or less than four scores, respectively.

### Statistical analysis

All statistical analyses in the cross-sectional study were performed with SPSS software, version 19 (IBM, USA). The continuous variable was presented as mean ± SD, and Student’s *t*-test was utilized to evaluate the difference between GSD group and non-GSD group. The categorical variable was expressed as number and percentage, and was compared by the Chi-square test. Multivariate logistic regression models were applied to evaluate the associations between serum lipids profile and GSD risk in each hospital. The effects of higher levels and each mmol/L increase in blood lipid levels on GSD were assessed. Finally, the results of the associations between serum lipid levels and GSD risk from the three hospitals were pooled. Subgroup analyses were conducted based on the subtype of GSD, age, and gender.

The STATA software (version 15, Stata, College Station, USA) was utilized for meta-analyses. Cochran’s *Q* test was performed to examine the heterogeneity between publications, and the *I*^*2*^ statistic was applied for quantification of heterogeneity. DerSimonian and Laird random-effect model was utilized when the heterogeneity was evident (*I*^*2*^ ≥ 50%); otherwise, fixed-effect model was adopted. For the included studies that provide the mean levels of blood lipids and SD, meta-analysis for continuous variables was conducted to obtain standard mean difference (SMD). For the studies that provide the estimates of lipid levels on GSD with OR/HR/RR and 95%CI, meta-analyses were performed on the effects of higher lipid levels or each increasing unit of blood lipid levels on GSD to obtain the pooled ORs and 95%CI. Next, a dose-response meta-analysis was conducted by robust error meta-regression (REMR) model using valid data [14, 15]. Three random knots were set based on the dose distribution’s quartiles. Studies with at least two categories of blood concentration were included. When the median concentrations of blood lipids were unavailable, the midpoint levels were used. Subgroup analyses were conducted based on geographic background, gender, study design, and quality grade. Meta-regression was also used to trace the source of heterogeneities. In addition, funnel plot and Begg’s test were performed to examined potential publication bias, while Egger’s test was used to explore small-study bias. Sensitivity analysis was conducted to verify the stability of the associations. Tests of heterogeneity and bias were one-tailed and *P*-value< 0.10 was deemed significant as recommended. The significance level of a two-tailed test was set at *P*-value less than 0.05.

## Results

### Baseline characteristics of the recruited subjects

Overall, 45,392 GSD cases and 503,542 controls were recruited from three hospitals in this multi-center cross-sectional study. The clinical, anthropometric characteristics and laboratory test results of the subjects are presented in Table 1. In the First Affiliated Hospital of Chongqing Medical University Jinshan Hospital, 170,038 subjects were enrolled and 12,518 (7.36%) of them had GSD. In the People’s Hospital of Kaizhou, a total of 372,289 subjects were recruited. GSD was found in 32,367 (8.69%) individuals. In Tianjin Medical University Cancer Institute and Hospital, 10,940 subjects were recruited, and 507 (4.63%) were diagnosed with GSD. The mean concentrations of TC, triglyceride, LDL cholesterol were remarkably higher in GSD cases, whereas HDL cholesterol was lower than in healthy controls. Compared to non-GSD group, GSD group also had significantly higher mean age and higher levels of FBG, SBP, DBP, AST, UN, and Cr. The prevalence rates of fatty liver disease, kidney stones, and hypertension in cases were higher than those in controls (all *P* < 0.05).

**Table 1:** Baseline characteristics of recruited subjects in each hospital according to gallstone disease status

	First Affiliated Hospital of Chongqing Medical University Jinshan Hospital (***n*** = 170,038)			The People’s Hospital of Kaizhou District of Chongqing (***n*** = 372,289)			Tianjin Medical University Cancer Institute and Hospital (***n*** = 10,940)		
	GSD (***n*** = 12,518)	Non-GSD (***n*** = 157,520)	Statistic	GSD (***n*** = 32,367)	Non-GSD (***n*** = 339,922)	Statistic	GSD (***n*** = 507)	Non-GSD (*n* = 10,433)	Statistic
**Age (year)**	51.70 ± 13.48	41.00 ± 13.14	− 87.54^**^	52.01 ± 12.34	43.20 ± 12.96	− 122.02^**^	55.69 ± 13.76	42.88 ± 13.35	−21.08^**^
**BMI (kg/m**^**2**^**)**	24.54 ± 3.40	23.23 ± 3.43	−39.28^**^	25.18 ± 3.27	23.85 ± 3.46	−67.61^**^	NA		
**WC (cm)**	84.43 ± 13.02	80.44 ± 27.62	−15.15^**^	85.52 ± 9.69	81.74 ± 10.18	−64.69^**^	NA		
**DBP (mmHg)**	78.16 ± 12.08	74.48 ± 11.44	−31.29^**^	79.43 ± 12.69	76.88 ± 12.24	−32.48^**^	80.60 ± 12.20	76.49 ± 11.75	−7.49^**^
**SBP (mmHg)**	129.86 ± 19.48	122.75 ± 17.17	−37.66^**^	128.96 ± 19.67	122.84 ± 17.82	−50.38^**^	139.42 ± 20.81	127.44 ± 18.91	−12.40^**^
**FBG (mmol/L)**	5.81 ± 1.58	5.39 ± 1.11	−29.20^**^	5.99 ± 1.75	5.54 ± 1.27	−44.24^**^	6.37 ± 1.98	5.63 ± 1.15	−8.24^**^
**AST (U/L)**	23.91 ± 18.78	23.12 ± 15.02	−5.43^**^	26.63 ± 17.06	25.87 ± 16.01	−8.02^**^	20.58 ± 14.70	18.81 ± 8.85	−2.68^*^
**ALT (U/L)**	27.93 ± 25.21	26.63 ± 25.66	−5.41^**^	28.49 ± 26.01	27.14 ± 24.76	−9.20^**^	31.13 ± 24.61	26.95 ± 22.47	−4.05^**^
**T-bil (umol/L)**	13.89 ± 5.67	13.65 ± 5.37	−4.61^**^	15.28 ± 6.72	15.01 ± 5.97	−4.23^**^	16.88 ± 5.60	17.02 ± 5.55	0.56
**Cr (umol/L)**	141.17 ± 944.41	102.29 ± 651.11	−4.46^**^	69.59 ± 19.27	70.8 ± 18.28	10.61^**^	80.17 ± 10.07	78.97 ± 11.29	−2.34^*^
**UA (umol/L)**	347.66 ± 92.64	345.08 ± 96.22	−2.95^*^	341.92 ± 88.75	341.65 ± 91.32	−0.48	313.79 ± 79.39	303.33 ± 82.77	−2.78^*^
**UN (mmol/L)**	5.28 ± 1.45	5.08 ± 1.30	−15.30^**^	5.32 ± 1.47	5.19 ± 1.42	−15.30^**^	4.63 ± 1.20	4.41 ± 1.17	−4.25^**^
**TC (mmol/L)**	5.02 ± 1.00	4.88 ± 0.94	−15.26^**^	5.14 ± 1.03	5.02 ± 0.99	−20.39^**^	5.59 ± 1.04	5.35 ± 1.01	−5.22^**^
**TG (mmol/L)**	1.91 ± 1.84	1.58 ± 1.43	−19.63^**^	2.01 ± 1.86	1.71 ± 1.58	−27.74^**^	1.55 ± 0.92	1.33 ± 0.93	−5.25^**^
**LDL-C (mmol/L)**	3.04 ± 0.84	2.92 ± 0.81	−15.41^**^	2.05 ± 0.62	1.97 ± 0.63	−18.69^**^	NA		
**HDL-C (mmol/L)**	1.36 ± 0.33	1.41 ± 0.34	15.35^**^	1.36 ± 0.37	1.40 ± 0.37	17.31^**^	NA		
**Female**	6121 (48.9%)	70,846 (45.0%)	71.99^**^	18,143 (56.1%)	155,377 (45.7%)	1270.80^**^	265 (52.3%)	5861 (56.2%)	3.00
**FLD**	5162 (41.2%)	37,795 (24.0%)	1826.04^**^	13,261 (41.0%)	87,569 (25.8%)	3457.16^**^	274 (54.0%)	3180 (30.5)	124.26^**^
**Kidney stones**	679 (5.4%)	6279 (4.0%)	61.10^**^	1197 (3.7%)	10,475 (3.1%)	36.87^**^	22 (4.3%)	233 (2.3%)	9.42^*^
**Hypertension**	4169 (36.9%)	27,439 (19.6%)	1906.37^**^	9226 (32.7%)	62,264 (20.8%)	2130.90^**^	246 (50.9%)	2592 (26.3%)	139.7^**^
**Gallstones**	6173 (49.3%)	–		14,598 (45.1%)	–		361 (71.2%)	–	
**Cholecystectomy**	6345 (50.7%)	–		17,769 (54.9%)	–		146 (28.8%)	–	

Quantitative data are presented as the mean ± SD, and the statistic is Z-value. Qualitative data are presented as N (%), and the statistic is Chi-square. *GSD* gallstone disease, *BMI* body mass index, *WC* waist circumference, *DBP* diastolic blood pressure, *SBP* systolic blood pressure, *FBG* fasting blood glycose, *AST* aspartate transaminase, *ALT* alanine transaminase, *T-bil* total bilirubin, *Cr* creatinine, *UA* uric Acid, *UN* urea nitrogen, *TC* total cholesterol, *TG* triglycerides, *LDL* low density lipoprotein cholesterol, *HDL* high density lipoprotein cholesterol, *FLD* Fatty liver disease. ^*^P < 0.05; ^**^ *P* < 0.001

### Multivariate logistic regression analysis of the associations between the concentrations of lipids and GSD risk in the three hospitals

Multivariate logistic regression analysis revealed that the higher levels of HDL cholesterol, LDL cholesterol and TC were negatively related to GSD risk in the two hospitals in Chongqing (Table 2). The higher level of triglyceride was not associated with GSD risk. However, for 1 mmol/L rise in triglyceride level, GSD risk was elevated. In Tianjin Medical University Cancer Institute and Hospital, multivariate analysis indicated GSD risk was not associated with TC and triglyceride. However, the results of subgroup analyses by age, gender, and diagnosis of GSD (Additional files 2, 3, 4) were inconsistent.

**Table 2:** Association between lipid profiles and gallstone disease with multivariate analysis by logistic regression

	First Affiliated Hospital of Chongqing Medical University Jinshan Hospital		The People’s Hospital of Kaizhou District of Chongqing		Tianjin Medical University Cancer Institute and Hospital		Pooled	
	OR (95%CI)	***P***	OR (95%CI)	***P***	OR (95%CI)	***P***	OR (95%CI)	***P***
**TC, mmol/L**								
< 3.1	ref		ref				ref	
3.1–5.7	**0.714 (0.599, 0.852)**	**< 0.001**	0.857 (0.688, 1.068)	0.170	ref		**0.767 (0.668, 0.880)**	**1.51 × 10**^**− 04**^
> 5.7	**0.670 (0.557, 0.807)**	**< 0.001**	**0.765 (0.608, 0.964)**	**0.023**	0.855 (0.701, 1.043)	0.122	**0.754 (0.671, 0.848)**	**2.25** × **10**^**−06**^
**TG, mmol/L**								
< 0.4	ref		ref				ref	
0.4–1.7	0.755 (0.524, 1.088)	0.132	1.089 (0.476, 2.493)	0.840	ref		0.801 (0.574, 1.119)	0.194
> 1.7	0.897 (0.621, 1.296)	0.562	1.272 (0.555, 2.916)	0.570	0.982 (0.784, 1.230)	0.874	0.972 (0.806, 1.172)	0.764
**LDL-C, mmol/L**								
< 2.07	ref		ref				ref	
2.07–3.1	0.932 (0.867, 1.002)	0.055	**0.902 (0.849, 0.958)**	**0.001**	NA		**0.914 (0.873, 0.958)**	**1.51 × 10**^**−04**^
> 3.1	**0.882 (0.815, 0.954)**	**0.002**	**0.905 (0.831, 0.985)**	**0.021**	NA		**0.893 (0.842, 0.946)**	**1.31 × 10**^**−04**^
**HDL-C, mmol/L**								
< 0.9	ref		ref				ref	
0.9–2.0	**0.906 (0.824, 0.996)**	**0.041**	0.915 (0.822, 1.019)	0.106	NA		**0.910 (0.848, 0.977)**	**0.009**
> 2.0	**0.698 (0.605, 0.804)**	**< 0.001**	**0.723 (0.623, 0.84)**	**< 0.001**	NA		**0.710 (0.640, 0.787)**	**7.03 × 10**^**−11**^
**TC, per unit**	**0.889 (0.827, 0.955)**	**0.001**	**0.881 (0.829, 0.937)**	**< 0.001**	0.939 (0.853, 1.033)	0.197	**0.895 (0.858, 0.933)**	**1.89 × 10**^**−07**^
**TG, per unit**	**1.054 (1.033, 1.075)**	**< 0.001**	**1.05 (1.031, 1.07)**	**< 0.001**	0.960 (0.862, 1.070)	0.461	**1.050 (1.036, 1.065)**	**8.68 × 10**^**−13**^
**LDLC, per unit**	1.071 (0.994, 1.153)	0.071	1.071 (0.988, 1.161)	0.094	NA		**1.071 (1.014, 1.131)**	**0.014**
**HDLC, per unit**	**0.832 (0.750, 0.922)**	**< 0.001**	0.930 (0.855, 1.012)	0.091	NA		**0.889 (0.833, 0.949)**	**4.32 × 10**^**−04**^

The ORs were adjusted for age, sex, BMI, fatty liver disease, kidney stone, hypertension, FBG, Cr, UA, UN, T-bil, ALT, AST, and TC, TG, LDL-C, LDL-C. Bold means *p* < 0.05, TC: total cholesterol, *TG* triglycerides, *LDL-C* low density lipoprotein cholesterol, *HDL-C* high density lipoprotein cholesterol

**Fig. 1:**
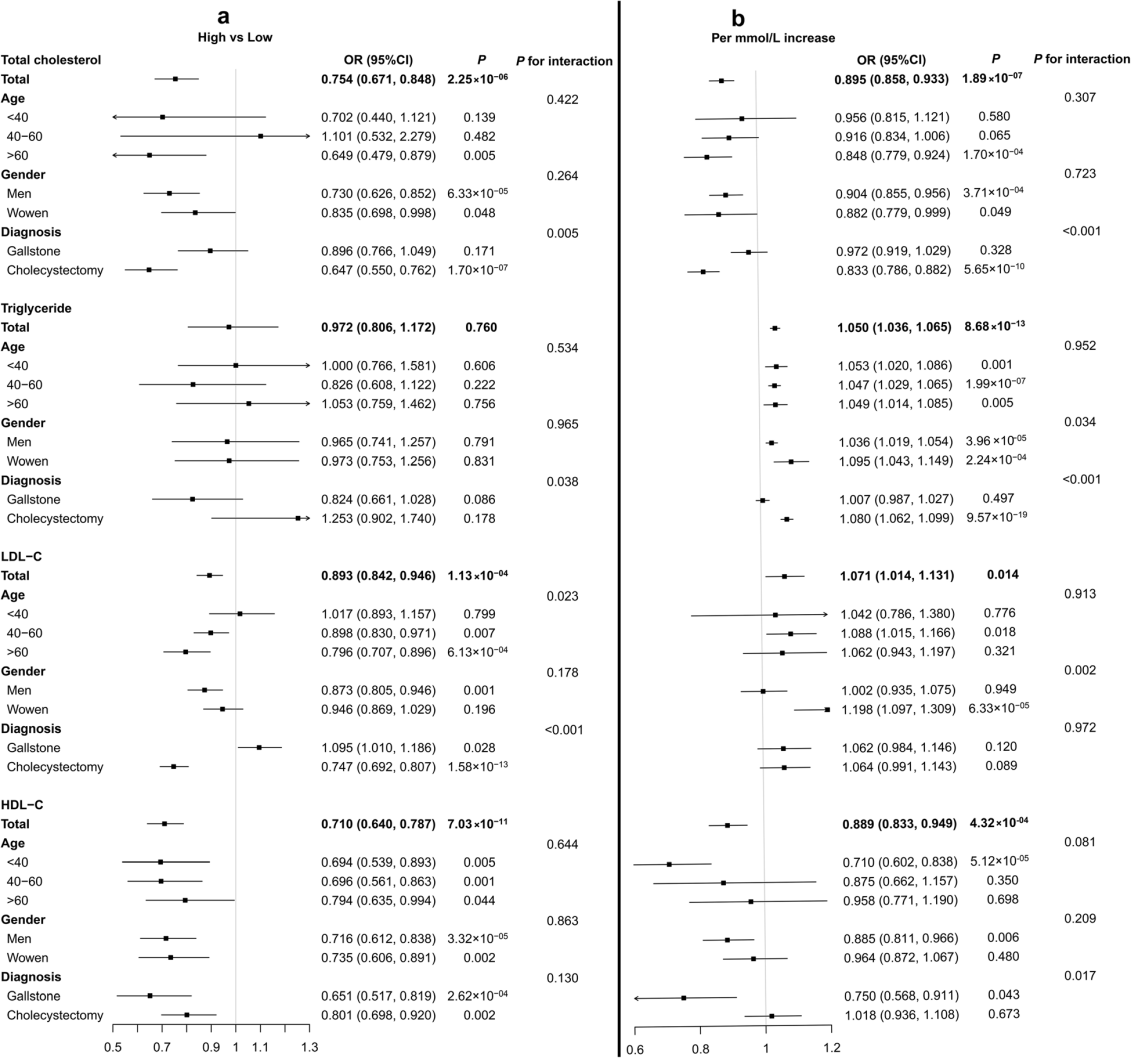
Pooled analysis of the multivariate logistic regression analysis in the multi-center cross-sectional study. **a** High-level vs Low-level model. **b** Per mmol/L increase model

Then, the results of each hospital were pooled. As shown in Table 2 and Fig. 1, the pooled analyses showed that for one unit raise in TC, the susceptibility of GSD was decreased by 10.5%. Compared to subjects with TC level of < 0.4 mmol/L, the GSD risks of those with TC levels of 3.1–5.7 and > 5.7 mmol/L were decreased by 23.3 and 24.6%, respectively. As to HDL cholesterol, the GSD risk was reduced by 11.1% for 1 mmol/L increase. Compared to the subjects with HDL cholesterol level of < 0.9 mmol/L, the GSD risks of those with HDL cholesterol levels of 0.9–2.0 and > 2.0 mmol/L were decreased by 9.0 and 29.0%, respectively. For LDL cholesterol, compared with the subjects with LDL cholesterol < 2.07 mmol/L, the GSD risk of those with LDL cholesterol levels of 2.07–3.1 and > 3.1 mmol/L were reduced by 8.6 and 10.7%, respectively. However, the results were not consistent with those in the model of each increase mmol/L in LDL cholesterol. The pooled results also demonstrated that, with one unit rise in triglyceride levels, the susceptibility of GSD was elevated by about 1.050 times. However, when considering triglyceride levels as a categorical variable, the GSD risk was not significantly increased when compared with participants with < 0.4 mmol/L.

When cholecystectomy and gallstones were analyzed separately, the higher concentrations of TC and LDL cholesterol were negatively related to cholecystectomy, but LDL cholesterol was related to a high susceptibility to gallstones formation. The high level of TC was associated with GSD risk only in the older population when stratified by age. Additionally, a higher level of LDL cholesterol was positively correlated with GSD risk in younger population, while negatively correlated with GSD risk in middle-aged and elderly populations (Fig. 1a). One mmol/L increase in triglyceride was positively associated with GSD risk when stratified by age and gender of the participants, but it was not associated with gallstone risk (Fig. 1b).

### Meta-analysis of the associations between blood lipid metabolism and GSD risk

**Fig. 2:**
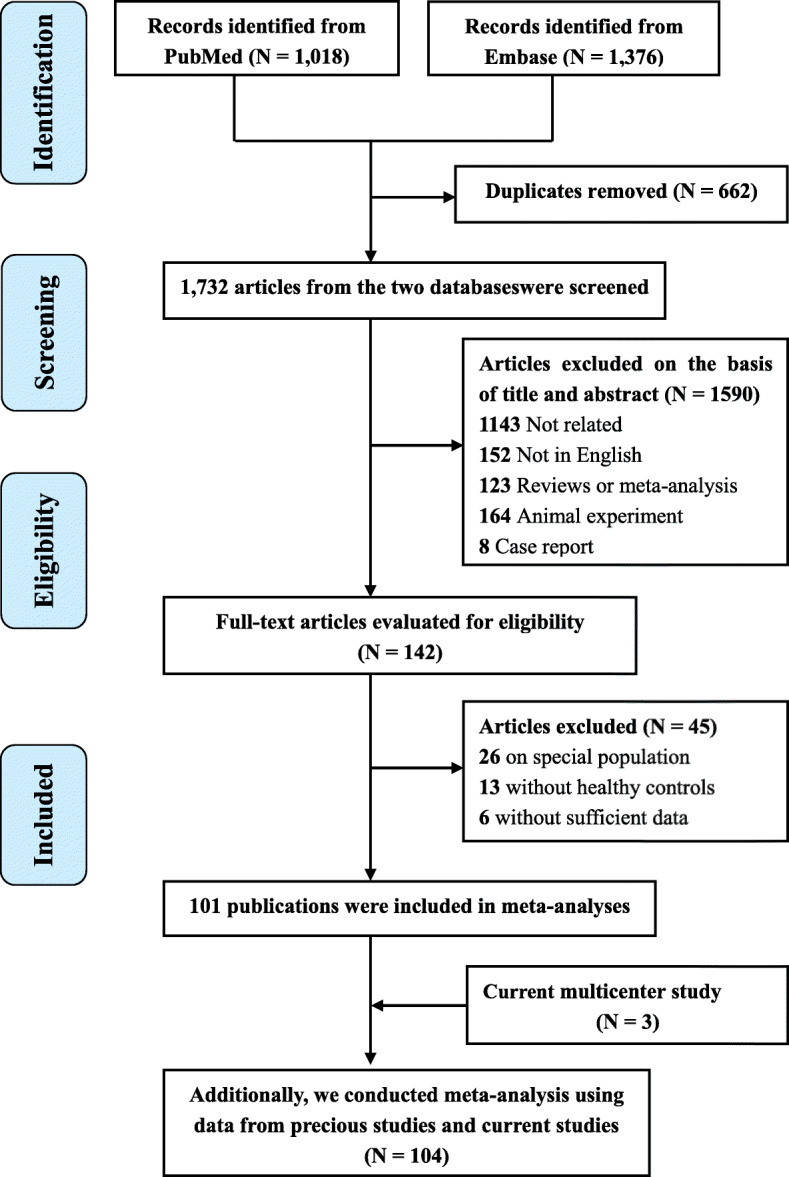
Flow-chart of the study selection in the present meta-analysis

The flowchart of literature selection process is presented in Fig. 2. This meta-analysis finally included 101 studies comprising 206,413 cases and 2,670,803 controls through screening a total of 1728 publications from the Medline and Embase databases. The characteristics of the included publications are shown in Additional files 5, 6. Of these studies, 74 reported the mean difference of blood lipid levels in GSD and non-GSD groups (Additional file 5), 27 reported estimates for an increasing unit in blood lipid levels, and 29 reported estimates for high-level versus low-level blood lipids (Additional file 6). There were 11, 33 and 57 cohort, case-control and cross-sectional studies, respectively. A majority of studies were carried out in Asian countries (*n* = 57), followed by European countries (*n* = 21), American countries (*n* = 18), Oceania country (*n* = 1), and other countries (*n* = 4). Among these studies, 41.6% (*n* = 42) were regarded as high quality and 58.4% (*n* = 59) were moderate quality according to the statement of NOS and AHRQ (Additional file 7). After adding three cross-sectional studies, 104 articles were finally included for the quantitative meta-analysis.

As shown in Table 3, meta-analyses of previous studies displayed that the mean concentrations of triglycerides and LDL cholesterol were markedly higher in GSD patients than in healthy individuals, but the concentration of HDL cholesterol was significantly lower, which is consistent with the current cross-sectional study. Meta-analyses showed that triglyceride was positively associated with GSD risk (ORs = 1.214 and 1.007 in high vs. low model and per unit model, respectively), while HDL cholesterol was negatively related to GSD risk (ORs = 0.625 and 0.978 in high vs. low model and per unit model, respectively) in the two comparison models. However, both TC nor LDL cholesterol were not significantly correlated with GSD risk.

**Table 3:** Relationship between blood lipid levels and gallstone disease risk in the meta-analysis

Lipids	Datasets	Present cross-sectional study				Previous studies					
		Cases	Controls	SMD (95%CI)	***P***	Datasets	Cases	Controls	SMD (95%CI)	***P***	***І***^***2***^
**TC**	3	45,392	507,875	0.148 (0.130, 0.166)	5.15 × 10^−18^	82	185,077	2,125,345	0.031 (−0.017, 0.079)	0.201	91.2%
**TG**	3	45,392	507,875	0.209 (0.174, 0.243)	1.70 × 10^−32^	78	183,975	2,077,581	0.390 (0.302, 0.477)	2.33 × 10^− 18^	97.6%
**LDL-C**	2	44,885	497,442	0.136 (0.116, 0.156)	1.36 × 10^−40^	56	181,859	2,081,769	0.070 (0.015, 0.125)	0.012	91.8%
**HDL-C**	2	44,885	497,442	−0.127 (−0.166, −0.089)	9.17 × 10^−11^	73	185,585	2,124,631	−0.215 (− 0.271, − 0.160)	2.54 × 10^−14^	94.0%
		**Cases**	**Controls**	**OR (95%CI)**	***P***	**Datasets**	**Cases**	**Controls**	**OR (95%CI)**	***P***	
**High vs Low**											
**TC**	3	45,392	507,875	0.754 (0.671, 0.848)	2.25 × 10^−06^	31	181,627	2,099,042	1.013 (0.929, 1.104)	0.768	72.7%
**TG**	3	45,392	507,875	0.972 (0.806, 1.172)	0.764	34	186,393	2,329,707	1.214 (1.111, 1.326)	1.71 × 10^−05^	77.7%
**LDL-C**	2	44,885	497,442	0.893 (0.842, 0.946)	1.13 × 10^−04^	19	176,698	2,056,688	1.072 (0.889, 1.292)	0.702	91.1%
**HDL-C**	2	44,885	497,442	0.710 (0.640, 0.787)	7.03 × 10^−11^	28	184,929	2,302,029	0.625 (0.552, 0.708)	1.58 × 10^−13^	85.8%
**Per unit**											
**TC**	3	45,392	507,875	0.895 (0.858, 0.933)	1.89 × 10^−07^	19	5478	100,853	0.999 (0.989, 1.010)	0.873	
**TG**	3	45,392	507,875	1.050 (1.036, 1.065)	8.68 × 10^−13^	25	6217	75,537	1.007 (1.002, 1.012)	0.010	91.1%
**LDL-C**	2	44,885	497,442	1.071 (1.014, 1.131)	0.014	15	5158	96,975	0.996 (0.987, 1.006)	0.487	
**HDL-C**	2	44,885	497,442	0.889 (0.833, 0.949)	4.32 × 10^−04^	25	8209	125,966	0.978 (0.965, 0.990)	4.32 × 10^−04^	75.3%
**Present and previous studies**											
	**Datasets**	**Cases**	**Controls**	**SMD (95%CI)**	***P***	***І***^***2***^	**Begg-*P***	**Egger-*P***			
**TC**	85	230,469	2,633,220	0.040 (0.009, 0.071)	0.012	90.9%	0.144	0.004			
**TG**	82	229,367	2,585,456	0.302 (0.251, 0.354)	1.32 × 10^−30^	97.7%	0.012	0.048			
**LDL-C**	59	226,744	2,579,211	0.059 (0.027, 0.092)	3.85 × 10^−04^	91.6%	0.024	0.014			
**HDL-C**	76	230,470	2,622,073	−0.182 (− 0.217, − 0.147)	3.67 × 10^−24^	93.9%	0.427	0.133			
	**Datasets**	**Cases**	**Controls**	**OR (95%CI)**	***P***	***І***^*2*^	**Begg-*P***	**Egger-*P***			
**High vs Low**											
**TC**	34	227,019	2,606,917	0.974 (0.896, 1.059)	0.539	76.5%	0.700	0.265			
**TG**	37	231,785	2,837,582	1.192 (1.097, 1.295)	3.47 × 10^−05^	75.8%	0.381	0.001			
**LDL-C**	21	221,583	2,554,130	1.054 (0.912, 1.219)	0.473	93.0%	0.651	0.749			
**HDL-C**	30	229,814	2,799,471	0.636 (0.570, 0.710)	5.97 × 10^−16^	85.0%	0.972	0.003			
**Per unit**											
**TC**	23	55,203	685,076	0.992 (0.981, 1.004)	0.177	84.5%	0.205	0.034			
**TG**	28	51,609	583,412	1.011 (1.006, 1.016)	5.12 × 10^−05^	96.0%	0.621	0.016			
**LDL-C**	18	54,376	670,765	0.998 (0.987, 1.008)	0.641	82.2%	0.829	0.899			
**HDL-C**	27	53,094	623,408	0.974 (0.961, 0.987)	6.07 × 10^−05^	76.4%	0.016	< 0.001			

*SMD* standard mean difference, *TC* total cholesterol, *TG* triglyceride, *LDL* low density lipoprotein cholesterol, *HDL* high density lipoprotein cholesterol

After adding the present multi-center study, the pooled results were relatively consistent (Table 3). Subgroup analyses (Additional file 8**)** also indicated that the associations were generally consistent when stratified by gender, study design, the grade of the quality and geographic background. The heterogeneity was significant in most of the meta-analysis models, and meta-regression showed that the study design, gender, geographic background, and quality of the included studies might be the sources of heterogeneity (Additional file 8). Begg’s test (Table 3) and funnel plot (Additional file 9) showed three associations with potential publication bias (*P* < 0.10). Egger’s test indicated eight associations with small-study bias (Table 3). However, sensitivity analysis showed that most of the associations did not alter significantly after excluding individual datasets (Additional file 10).

As to the dose-response meta-analysis, a total of 17, 18, 12 and 15 studies reporting on the associations between GSD risk and levels of TC, triglycerides, LDL cholesterol, HDL cholesterol were included, respectively, according to the criteria of the REMR model. The doses ranged from 0 to 15.22, 0 to 4.47, 0 to 7.41, and 0 to 2.85 for TC, triglycerides, LDL cholesterol, and HDL cholesterol, respectively. A linear relationship between HDL cholesterol level and GSD risk (*P*-value for nonlinearity = 0.1562) was observed (Additional files 11 and 12). In contrast, nonlinear relationships between TC (*P*-value for nonlinearity = 0.0003), triglycerides (*P*-value for nonlinearity = 0.0005), LDL cholesterol (*P*-value for nonlinearity = 0.0021) and GSD risk were detected.

## Discussion

This study comprehensively investigated the associations between blood lipid metabolism and GSD risk by combining multi-center cross-sectional research and meta-analysis. The combined results showed triglyceride was positively associated with GSD risk, while HDL cholesterol was negatively related to GSD risk. However, the levels of TC and LDL cholesterol were not remarkably associated with GSD risk.

The exact role of serum lipid levels in GSD remains unclear. The involvement of lipids in some critical lithogenic processes of gallstones might be a possible explanation for their effects on GSD risk. Generally, the mechanisms of cholesterol gallstone formation depend on the cholesterol crystals in bile, which is related to an increasing bile cholesterol saturation index and is negatively correlated to the levels of bile salts. Furthermore, the time of cholesterol crystal nucleation and dysfunction of the gallbladder also affect the formation of gallstones [16].

The present study found that HDL cholesterol was negatively related to GSD risk. This result is in agreement with previous epidemiologic studies [2, 8, 17] reporting on the inverse association between HDL cholesterol and GSD. Evidence has shown that a high level of blood HDL cholesterol can facilitate the synthesis of hepatic bile acid [18] and decrease the cholesterol saturation index [19], which in turn increases cholesterol solubility in the bile [20] and subsequently protects against gallstones formation. Alternatively, it has been reported that HDL cholesterol contributes most of the cholesterol transported into bile [21]. Thus, given the negative correlation between HDL cholesterol and GSD risk observed in this research, it can be inferred that the free cholesterol in HDL is preferentially metabolized to bile acid rather than secreted into the bile as cholesterol.

In contrast to HDL cholesterol, this meta-analysis showed that triglyceride was positively related to GSD susceptibility. This conclusion is consistent with previous cohort studies [8, 22]. However, the mechanisms underlying the increased levels of triglyceride in patients with GSD are not clear. The findings by Cavallini et al. demonstrated that a high level of serum triglyceride was positively related to an increase in cholesterol saturation index [23] and rapid nucleation of cholesterol crystals [18], which were key precursors for gallstone formation.

As for the association between TC, LDL cholesterol and GSD risk, the results from previous researches was quite conflicting, with some observational studies reporting no association [8, 17] and other researchers considered TC and LDL cholesterol as protective [24] or risk factors for GSD [2, 25]. This multi-center cross-sectional study indicated that the high level of TC was negatively associated with GSD only in older individuals and that high LDL cholesterol level was related to a decreased susceptibility of GSD among the middle-aged and older-aged groups. This finding indicates that age is an important factor to consider, and provides a sensible explanation for the seeming paradox that the mean levels of TC and LDL cholesterol were remarkably higher in GSD cases than in controls, but negatively associated with GSD risk after adjusting for multiple factors in the cross-sectional study. Furthermore, subgroup analyses added weight to the speculation that the inconsistencies might arise from variations in the study population, study design, lipid measurement methods, or mixed control deficiencies. The mechanism of how TC and LDL cholesterol are implicated in GSD remains elusive; thus, further functional experiments are warranted.

Another indispensable factor to consider is sex, which has been widely accepted as a risk factor for GSD [3]. Subgroup analyses in the meta-analysis showed that GSD risk was positively related to triglyceride in females but not in males, and this result is in good agreement with previous findings [26]. In addition, it was found that TC, HDL cholesterol and LDL cholesterol had stronger protective effects in females than in males. Observational studies have indicated that low levels of testosterone are negatively associated with triglyceride, but TC and LDL cholesterol were positively related to HDL cholesterol in men [27]. Besides, it has been proposed that estrogens increase the risk of GSD by upregulating the hepatic secretion of biliary cholesterol, leading to a rise in the cholesterol saturation of bile [28]. Estrogen administration could also increase and decrease triglyceride and LDL cholesterol, respectively [29], suggesting that sex hormones are involved in the associations between blood lipids and GSD.

Evidence has shown that patients who underwent cholecystectomy might have different serum lipid profiles compared to those with gallstones [30, 31]. In this study, TC and LDL cholesterol appeared to be negatively associated with cholecystectomy, but not positively associated with gallstones. Consistent with this, some studies reported that GSD patients who underwent cholecystectomy had decreased levels of TC and triglyceride and increased level of HDL cholesterol [30], suggesting that cholecystectomy can improve blood lipid levels. In contrast, Chacez-Tapia et al. [31] showed that GSD patients after cholecystectomy had higher levels of LDL cholesterol, triglyceride and TC, but a lower concentration of HDL cholesterol than controls. The bile in the liver directly enters the intestine after cholecystectomy, resulting in faster bile acid circulation and thus exposing the enterohepatic system to a faster bile acid flux. Bile acid and lipid metabolisms are functionally inter-related [32]. Therefore, more efforts should be made to further understand the exact role of cholecystectomy on lipid metabolism.

Genetic factors may also contribute to the relationship between blood lipids and GSD risk. It has been suggested that Q6404 and D19H polymorphisms in the ATP binding cassette protein G5/G8 (*ABCG5/G8*) genes are significantly related to higher concentrations of triglyceride and lower concentrations of HDL cholesterol [33]. Other genetic studies have also identified that the variants in *ABCG5/G8* genes are associated with the risk of GSD [34]. *ABCG5/G8* gene products function as a half-transporter to promote cholesterol transport into bile. In patients with GSD, the expression of *ABCG5/G8* was upregulated and could affect the cholesterol supersaturation of bile [35]. Besides, rare mutations in cytochrome P450 family 7 subfamily A member 1 (*CYP7A1*) gene also result in premature GSD and hypertriglyceridemia [36], and the product of *CYP7A1* gene catalyzes the initial steps of cholesterol catabolism and cholic acid synthesis.

### Study strengths and limitations

The main strength of this study is that it provides the most detailed assessment to date on the contribution of serum lipid profiles to GSD. Based on the multi-center cross-sectional study and meta-analysis with approximately 3 million participants, the association between blood lipid metabolism and GSD risk was comprehensively evaluated. However, this study has several limitations. First, problems inherent in the design of cross-sectional study should be noticed, and re-assessment of prospective studies may be helpful to further elucidate the role of lipids in gallstone formation. Second, it was unable to define a unified analytical standard across the included studies since the raw data could not be obtained from all studies. To minimize bias, the adjusted risk estimates were extracted and the risk estimates under the high vs. low level model or per increasing unit model were adopted. Third, some of the associations in meta-analysis showed significant heterogeneity and bias. Meta-regression analysis indicated that study design, gender, geographic background, and quality of the included studies might be the sources of heterogeneity. Other factors, such as the adjustment of ORs, could also lead to statistical heterogeneity. Sensitivity analysis indicated that only one of the associations was changed after excluding individual datasets.

## Conclusions

In conclusion, this multi-center cross-sectional study and meta-analysis provide updated extensive evidence on the correlations between blood lipid metabolism and GSD risk. The results imply that low HDL cholesterol levels and high triglyceride levels are risk factors for GSD. This study provides a basis for identifying the population at high risk for GSD and also a possible way for the prevention and control of GSD, that is, implementing preventive intervention for patients with high and low concentrations of triglyceride and HDL cholesterol, respectively. Further preventive intervention trials are needed to verify the effects of triglyceride and HDL cholesterol on GSD.

## Supplementary Information

**Additional file 1.** Search terms for the electronic literature database search in the meta-analysis.

**Additional file 2.** Associations between blood lipid levels and gallstones or cholecystectomy in our cross-sectional study in each hospital.

**Additional file 3.** Subgroup analysis for relationships between blood lipid profiles and gallstone disease by age group in our cross-sectional study.

**Additional file 4.** Subgroup analysis for relationships between blood lipid levels and gallstone disease by gender in our cross-sectional study.

**Additional file 5.** The characteristics of the included publications regarding the mean difference of the blood lipid levels between groups.

**Additional file 6.** The characteristics of the included publications regarding OR and 95%CI of the blood lipid levels on GSD.

**Additional file 7.** Quality assessment of the included studies.

**Additional file 8.** Subgroup analysis and meta-regression in meta-analysis.

**Additional file 9.** Funnel plots for the final meta-analyses. TC: total cholesterol, LDL-C: low-density lipoprotein cholesterol, HDL-C: high-density lipoprotein cholesterol, SD: standard deviation, OR: odd ratios.

**Additional file 10.** Sensitivity analysis for the final meta-analyses. TC: total cholesterol, LDL-C: low-density lipoprotein cholesterol, HDL-C: high-density lipoprotein cholesterol, SD: standard deviation, OR: odd ratios.

**Additional file 11.** Estimated regression parameters and standard errors in the dose-response meta-analysis.

**Additional file 12.** Dose-response relationships between GSD risk and levels of (A) total cholesterol; (B) LDL-C; (C) Triglycerides; (D) HDL-C.

## Data Availability

The datasets supporting the conclusions of this article are included within the article and its additional files.

## Abbreviations

GSD: gallstone disease
TC: total cholesterol
LDL cholesterol: low-density lipoprotein cholesterol
HDL cholesterol: high-density lipoprotein cholesterol
SE: standard error
SD: standard deviation
SMD: standard mean difference
