# Supplementary material for: Blood lipid metabolism and the risk of gallstone disease: a multi-center study and meta-analysis
Source: Lipids Health Dis. 2022 Mar 2;21:26. doi: 10.1186/s12944-022-01635-9 (PMC8889751; doi:10.1186/s12944-022-01635-9)
Supplement: Supplementary file 1 — Additional file 1. Search terms for the electronic literature database search in the meta-analysis. [file 12944_2022_1635_MOESM1_ESM.docx]

**Additional file 1.** Search terms for the electronic literature database search in the meta-analysis

|  | **Boolean connectives** | **Key words** |
| --- | --- | --- |
| **Medline database (A+B: N = 1018)** | | |
| **A** |  | ("gallstone"[Title/Abstract] OR "gallstone disease"[Title/Abstract] OR "gallbladder stone"[Title/Abstract] OR "gallbladder calculus"[Title/Abstract] OR "biliary tract stone"[Title/Abstract] OR "bile duct stone"[Title/Abstract] OR "choledocholithiasis"[Title/Abstract] OR "cholelithiasis"[Title/Abstract] OR "cholecystolithiasis"[Title/Abstract] OR "hepatolithiasis"[Title/Abstract]) |
| **B** | **AND** | ("lipid"[Title/Abstract] OR "blood lipid"[Title/Abstract] OR "total cholesterol"[Title/Abstract] OR "triglyceride"[Title/Abstract] OR "high density lipoprotein"[Title/Abstract] OR "HDL"[Title/Abstract] OR "hdl cholesterol"[Title/Abstract] OR "HDL-C"[Title/Abstract] OR "low density lipoprotein"[Title/Abstract] OR "LDL"[Title/Abstract] OR "ldl cholesterol"[Title/Abstract] OR "LDL-C"[Title/Abstract]) |
| **Embase database (A+B: N = 1376)** | | |
| **A** |  | (gallstone:ab,ti OR 'gallstone disease':ab,ti OR 'gallbladder stone':ab,ti OR 'gallbladder calculus':ab,ti OR 'biliary tract stone':ab,ti OR bile duct stone':ab,ti OR choledocholithiasis:ab,ti OR cholelithiasis:ab,ti OR cholecystolithiasis:ab,ti OR hepatolithiasis:ab,ti) |
| **B** | **AND** | (lipid:ab,ti OR 'blood lipid':ab,ti OR total cholesterol':ab,ti OR triglyceride:ab,ti OR 'high density lipoprotein':ab,ti OR hdl:ab,ti OR hdl cholesterol':ab,ti OR 'hdl c':ab,ti OR 'low density lipoprotein':ab,ti OR ldl:ab,ti OR 'ldl cholesterol':ab,ti OR 'ldl c':ab,ti) |
