# Supplementary material for: Blood lipid metabolism and the risk of gallstone disease: a multi-center study and meta-analysis
Source: Lipids Health Dis. 2022 Mar 2;21:26. doi: 10.1186/s12944-022-01635-9 (PMC8889751; doi:10.1186/s12944-022-01635-9)
Supplement: Supplementary file 2 — Additional file 2. Associations between blood lipid levels and gallstones or cholecystectomy in our cross-sectional study in each hospital. [file 12944_2022_1635_MOESM2_ESM.docx]

**Additional file 2.** Associations between blood lipid levels and gallstones or cholecystectomy in our cross-sectional study in each hospital

| **Subgroup** |  | **First Affiliated Hospital of Chongqing Medical University Jinshan Hospital** | | **The People’s Hospital of Kaizhou District of Chongqing** | | **Tianjin Medical University Cancer Institute and Hospital** | |
| --- | --- | --- | --- | --- | --- | --- | --- |
|  |  | **OR (95%CI)** | ***P*** | **OR (95%CI)** | ***P*** | **OR (95%CI)** | ***P*** |
| **Gallstones** | **TC, mmol/L** |  |  |  |  |  |  |
|  | <3.1 | Ref |  | Ref |  |  |  |
|  | 3.1-5.7 | 0.829 (0.645, 1.066) | 0.144 | 1.092 (0.770, 1.549) | 0.622 |  |  |
|  | >5.7 | 0.816 (0.627, 1.061) | 0.129 | 0.987 (0.686, 1.419) | 0.943 | 0.927 (0.735, 1.169) | 0.522 |
|  | **TG, mmol/L** |  |  |  |  |  |  |
|  | <0.4 | Ref |  | Ref |  |  |  |
|  | 0.4-1.7 | **0.549 (0.362, 0.831)** | **0.005** | 0.978 (0.310, 3.087) | 0.969 | Ref |  |
|  | >1.7 | **0.598 (0.393, 0.911)** | **0.017** | 1.029 (0.325, 3.256) | 0.961 | 0.926 (0.709, 1.208) | 0.570 |
|  | **LDL-C, mmol/L** |  |  |  |  |  |  |
|  | <2.07 | Ref |  | Ref |  |  |  |
|  | 2.07-3.1 | 0.994 (0.900, 1.099) | 0.913 | 0.988 (0.905, 1.079) | 0.792 |  |  |
|  | >3.1 | 1.064 (0.955, 1.185) | 0.261 | **1.134 (1.005, 1.279)** | **0.041** |  |  |
|  | **HDL-C, mmol/L** |  |  |  |  |  |  |
|  | <0.9 | Ref |  | Ref |  |  |  |
|  | 0.9-2.0 | **0.815 (0.718, 0.925)** | **0.001** | **0.846 (0.728, 0.983)** | **0.029** |  |  |
|  | >2.0 | **0.580 (0.475, 0.707)** | **<0.001** | **0.734 (0.594, 0.907)** | **0.004** |  |  |
|  |  |  |  |  |  |  |  |
|  | **TC, per unit** | 1.022 (0.925, 1.130) | 0.664 | 0.926 (0.849, 1.010) | 0.082 | 0.990 (0.885, 1.106) | 0.854 |
|  | **TG, per unit** | 1.006 (0.977, 1.036) | 0.692 | 1.012 (0.984, 1.040) | 0.403 | 0.910 (0.795, 1.043) | 0.176 |
|  | **LDLC, per unit** | 1.016 (0.917, 1.125) | 0.767 | **1.123 (1.002, 1.259)** | **0.046** |  |  |
|  | **HDLC, per unit** | **0.649 (0.560, 0.751)** | **<0.001** | **0.862 (0.761, 0.975)** | **0.018** |  |  |
| **Cholecystectomy** | **TC, mmol/L** |  |  |  |  |  |  |
|  | <3.1 | Ref |  | Ref |  |  |  |
|  | 3.1-5.7 | **0.664 (0.529, 0.834)** | **<0.001** | **0.739 (0.565, 0.967)** | **0.028** | 1 |  |
|  | >5.7 | **0.604 (0.475, 0.769)** | **<0.001** | **0.665 (0.501, 0.883)** | **0.005** | 0.720 (0.504, 1.029) | 0.071 |
|  | **TG, mmol/L** |  |  |  |  |  |  |
|  | <0.4 | Ref |  | Ref |  |  |  |
|  | 0.4-1.7 | 1.374 (0.677, 2.787) | 0.379 | 1.169 (0.368, 3.715) | 0.791 | Ref |  |
|  | >1.7 | 1.730 (0.851, 3.521) | 0.130 | 1.457 (0.458, 4.635) | 0.524 | 1.117 (0.756, 1.651) | 0.580 |
|  | **LDL-C, mmol/L** |  |  |  |  |  |  |
|  | <2.07 | Ref |  | Ref |  |  |  |
|  | 2.07-3.1 | **0.880 (0.798, 0.970)** | **0.010** | **0.845 (0.781, 0.913)** | **<0.001** |  |  |
|  | >3.1 | **0.744 (0.669, 0.828)** | **<0.001** | **0.751 (0.671, 0.840)** | **<0.001** |  |  |
|  | **HDL-C, mmol/L** |  |  |  |  |  |  |
|  | <0.9 | Ref |  | Ref |  |  |  |
|  | 0.9-2.0 | 1.016 (0.892, 1.158) | 0.810 | 0.984 (0.853, 1.136) | 0.826 |  |  |
|  | >2.0 | 0.864 (0.714, 1.047) | 0.136 | **0.738 (0.605, 0.901)** | **0.003** |  |  |
|  |  |  |  |  |  |  |  |
|  | **TC, per unit** | **0.799 (0.727, 0.878)** | **<0.001** | **0.858 (0.792, 0.930)** | **<0.001** | **0.835 (0.701, 0.995)** | **0.044** |
|  | **TG, per unit** | **1.088 (1.061, 1.115)** | **<0.001** | **1.073 (1.048, 1.099)** | **<0.001** | 1.065 (0.907, 1.249) | 0.444 |
|  | **LDLC, per unit** | **1.103 (1.001, 1.216)** | **0.049** | 1.020 (0.918, 1.135) | 0.709 |  |  |
|  | **HDLC, per unit** | 1.060 (0.927, 1.212) | 0.393 | 0.992 (0.890, 1.105) | 0.882 |  |  |

The ORs were adjusted for age, sex, BMI, fatty liver disease, kidney stone, hypertension, FBG, Cr, UA, UN, TBIL, ALT, AST, and TC, TG, LDL-C, LDL-C. Bold means *p* < 0.05, TC: total cholesterol, TG: triglycerides, LDL: low density lipoprotein cholesterol, HDL: high density lipoprotein cholesterol.
