# Supplementary material for: Blood lipid metabolism and the risk of gallstone disease: a multi-center study and meta-analysis
Source: Lipids Health Dis. 2022 Mar 2;21:26. doi: 10.1186/s12944-022-01635-9 (PMC8889751; doi:10.1186/s12944-022-01635-9)
Supplement: Supplementary file 3 — Additional file 3. Subgroup analysis for relationships between blood lipid profiles and gallstone disease by age group in our cross-sectional study. [file 12944_2022_1635_MOESM3_ESM.docx]

**Additional file 3**. Subgroup analysis for relationships between blood lipid profiles and gallstone disease by age group in our cross-sectional study

| **Subgroup** |  | **First Affiliated Hospital of Chongqing Medical University Jinshan Hospital** | | **The People’s Hospital of Kaizhou District of Chongqing** | | **Tianjin Medical University Cancer Institute and Hospital** | |
| --- | --- | --- | --- | --- | --- | --- | --- |
|  |  | **OR (95%CI)** | ***P*** | **OR (95%CI)** | ***P*** | **OR (95%CI)** | ***P*** |
| **Age <40** | **TC, mmol/L** |  |  |  |  |  |  |
|  | <3.1 | Ref |  | Ref |  |  |  |
|  | 3.1-5.7 | **0.529 (0.391, 0.717)** | **<0.001** | 0.976 (0.614, 1.552) | 0.918 | Ref |  |
|  | >5.7 | **0.493 (0.350, 0.694)** | **<0.001** | 1.000 (0.602, 1.661) | 0.999 | 0.786 (0.419, 1.474) | 0.452 |
|  | **TG, mmol/L** |  |  |  |  |  |  |
|  | <0.4 | Ref |  | Ref |  |  |  |
|  | 0.4-1.7 | 0.687 (0.444, 1.063) | 0.092 | 1.778 (0.436, 7.257) | 0.422 | Ref |  |
|  | >1.7 | 0.922 (0.587, 1.45) | 0.727 | 2.151 (0.524, 8.836) | 0.288 | 1.395 (0.713, 2.730) | 0.330 |
|  | **LDL-C, mmol/L** |  |  |  |  |  |  |
|  | <2.07 | Ref |  | Ref |  |  |  |
|  | 2.07-3.1 | 1.086 (0.952, 1.240) | 0.218 | 0.982 (0.863, 1.117) | 0.784 |  |  |
|  | >3.1 | 1.086 (0.926, 1.274) | 0.312 | 0.896 (0.718, 1.118) | 0.330 |  |  |
|  | **HDL-C, mmol/L** |  |  |  |  |  |  |
|  | <0.9 | Ref |  | Ref |  |  |  |
|  | 0.9-2.0 | 0.979 (0.797, 1.204) | 0.844 | 0.830 (0.646, 1.066) | 0.144 |  |  |
|  | >2.0 | 0.775 (0.560, 1.073) | 0.125 | **0.586 (0.392, 0.874)** | **0.009** |  |  |
|  | **TC, per unit** | 0.851 (0.724, 1.000) | 0.051 | 1.075 (0.916, 1.262) | 0.376 | 0.953 (0.712, 1.277) | 0.749 |
|  | **TG, per unit** | **1.067 (1.023, 1.113)** | **0.002** | 1.037 (0.989, 1.088) | 0.136 | 0.899 (0.594, 1.359) | 0.613 |
|  | **LDLC, per unit** | **1.194 (1.009, 1.412)** | **0.038** | 0.896 (0.727, 1.104) | 0.302 |  |  |
|  | **HDLC, per unit** | **0.738 (0.586, 0.929)** | **0.010** | **0.682 (0.538, 0.866)** | **0.002** |  |  |
| **Age 40-60** | **TC, mmol/L** |  |  |  |  |  |  |
|  | <3.1 | Ref |  | Ref |  |  |  |
|  | 3.1-5.7 | 0.737 (0.541, 1.004) | 0.053 | 1.122 (0.790, 1.595) | 0.520 | Ref |  |
|  | >5.7 | **0.704 (0.512, 0.968)** | **0.031** | 0.988 (0.688, 1.417) | 0.946 | 1.073 (0.814, 1.415) | 0.615 |
|  | **TG, mmol/L** |  |  |  |  |  |  |
|  | <0.4 | Ref |  | Ref |  |  |  |
|  | 0.4-1.7 | 1.338 (0.536, 3.339) | 0.533 | 0.787 (0.237, 2.616) | 0.696 | Ref |  |
|  | >1.7 | 1.554 (0.622, 3.886) | 0.346 | 0.907 (0.273, 3.016) | 0.873 | 0.753 (0.538, 1.056) | 0.100 |
|  | **LDL-C, mmol/L** |  |  |  |  |  |  |
|  | <2.07 | Ref |  | Ref |  |  |  |
|  | 2.07-3.1 | 0.917 (0.820, 1.024) | 0.123 | **0.868 (0.803, 0.939)** | **<0.001** |  |  |
|  | >3.1 | **0.876 (0.780, 0.983)** | 0.025 | 0.917 (0.824, 1.020) | 0.112 |  |  |
|  | **HDL-C, mmol/L** |  |  |  |  |  |  |
|  | <0.9 | Ref |  | Ref |  |  |  |
|  | 0.9-2.0 | **0.843 (0.745, 0.953)** | **0.006** | 0.941 (0.823, 1.075) | 0.369 |  |  |
|  | >2.0 | **0.622 (0.512, 0.755)** | **<0.001** | **0.775 (0.645, 0.930)** | **0.006** |  |  |
|  | **TC, per unit** | 0.923 (0.841, 1.014) | 0.094 | **0.851 (0.788, 0.918)** | **<0.001** | 1.015 (0.887, 1.162) | 0.824 |
|  | **TG, per unit** | **1.042 (1.015, 1.070)** | **0.002** | **1.053 (1.029, 1.077)** | **<0.001** | 0.927 (0.801, 1.073) | 0.310 |
|  | **LDLC, per unit** | 1.051 (0.955, 1.157) | 0.305 | **1.129 (1.021, 1.248)** | **0.018** |  |  |
|  | **HDLC, per unit** | **0.756 (0.657, 0.871)** | **<0.001** | 1.005 (0.908, 1.112) | 0.929 |  |  |
| **Age >60** | **TC, mmol/L** |  |  |  |  |  |  |
|  | <3.1 | Ref |  | Ref |  |  |  |
|  | 3.1-5.7 | 0.895 (0.663, 1.208) | 0.468 | **0.545 (0.371, 0.801)** | **0.002** |  |  |
|  | >5.7 | 0.833 (0.604, 1.149) | 0.265 | **0.475 (0.314, 0.718)** | **<0.001** |  |  |
|  | **TG, mmol/L** |  |  |  |  |  |  |
|  | <0.4 | Ref |  | Ref |  |  |  |
|  | 0.4-1.7 | 0.472 (0.164, 1.362) | 0.165 | 0.506 (0.042, 6.052) | 0.591 | Ref |  |
|  | >1.7 | 0.544 (0.188, 1.575) | 0.262 | 0.601 (0.050, 7.197) | 0.688 | **0.648 (0.466, 0.899)** | **0.009** |
|  | **LDL-C, mmol/L** |  |  |  |  |  |  |
|  | <2.07 | Ref |  | Ref |  |  |  |
|  | 2.07-3.1 | **0.822 (0.713, 0.947)** | **0.007** | 0.938 (0.813, 1.083) | 0.385 | Ref |  |
|  | >3.1 | **0.754 (0.648, 0.877)** | **<0.001** | 0.868 (0.716, 1.051) | 0.147 | 1.143 (0.807, 1.618) | 0.453 |
|  | **HDL-C, mmol/L** |  |  |  |  |  |  |
|  | <0.9 | Ref |  | Ref |  |  |  |
|  | 0.9-2.0 | 1.123 (0.902, 1.397) | 0.299 | 0.971 (0.744, 1.267) | 0.829 |  |  |
|  | >2.0 | 0.874 (0.656, 1.164) | 0.356 | **0.684 (0.478, 0.979)** | **0.038** |  |  |
|  | **TC, per unit** | **0.835 (0.714, 0.977)** | **0.025** | **0.873 (0.764, 0.998)** | **0.046** | **0.828 (0.706, 0.971)** | **0.020** |
|  | **TG, per unit** | **1.064 (1.013, 1.118)** | **0.013** | 1.038 (0.990, 1.089) | 0.122 | 1.008 (0.824, 1.233) | 0.940 |
|  | **LDLC, per unit** | 1.085 (0.923, 1.276) | 0.322 | 1.036 (0.868, 1.237) | 0.692 |  |  |
|  | **HDLC, per unit** | 1.072 (0.873, 1.316) | 0.510 | 0.859 (0.705, 1.047) | 0.133 |  |  |

The ORs were adjusted for age, sex, BMI, fatty liver disease, kidney stone, hypertension, FBG, Cr, UA, UN, TBIL, ALT, AST, and TC, TG, LDL-C, LDL-C. Bold means *p* < 0.05, TC: total cholesterol, TG: triglycerides, LDL: low density lipoprotein cholesterol, HDL:high density lipoprotein cholesterol.
