# Supplementary material for: Blood lipid metabolism and the risk of gallstone disease: a multi-center study and meta-analysis
Source: Lipids Health Dis. 2022 Mar 2;21:26. doi: 10.1186/s12944-022-01635-9 (PMC8889751; doi:10.1186/s12944-022-01635-9)
Supplement: Supplementary file 4 — Additional file 4. Subgroup analysis for relationships between blood lipid levels and gallstone disease by gender in our cross-sectional study. [file 12944_2022_1635_MOESM4_ESM.docx]

**Additional file 4.** Subgroup analysis for relationships between blood lipid levels and gallstone disease by gender in our cross-sectional study

| **Subgroup** |  | **First Affiliated Hospital of Chongqing Medical University Jinshan Hospital** | | **The People’s Hospital of Kaizhou District of Chongqing** | | **Tianjin Medical University Cancer Institute and Hospital** | |
| --- | --- | --- | --- | --- | --- | --- | --- |
|  |  | **OR (95%CI)** | ***P*** | **OR (95%CI)** | ***P*** | **OR (95%CI)** | ***P*** |
| **Male** | **TC, mmol/L** |  |  |  |  |  |  |
|  | <3.1 | Ref |  | Ref |  |  |  |
|  | 3.1-5.7 | **0.719 (0.577, 0.897)** | **0.003** | 0.838 (0.628, 1.120) | 0.233 | Ref |  |
|  | >5.7 | **0.643 (0.509, 0.811)** | **<0.001** | **0.737 (0.544, 0.999)** | **0.049** | 0.869 (0.658, 1.147) | 0.321 |
|  | **TG, mmol/L** |  |  |  |  |  |  |
|  | <0.4 | Ref |  | Ref |  |  |  |
|  | 0.4-1.7 | 0.705 (0.350, 1.421) | 0.329 | 0.395 (0.112, 1.390) | 0.148 | Ref |  |
|  | >1.7 | 0.824 (0.408, 1.663) | 0.589 | 0.447 (0.127, 1.575) | 0.210 | 1.034 (0.771, 1.385) | 0.825 |
|  | **LDL-C, mmol/L** |  |  |  |  |  |  |
|  | <2.07 | Ref |  | Ref |  |  |  |
|  | 2.07-3.1 | 0.929 (0.835, 1.032) | 0.170 | **0.907 (0.829, 0.993)** | **0.034** |  |  |
|  | >3.1 | **0.854 (0.765, 0.954)** | **0.005** | 0.895 (0.795, 1.007) | 0.066 |  |  |
|  | **HDL-C, mmol/L** |  |  |  |  |  |  |
|  | <0.9 | Ref |  | Ref |  |  |  |
|  | 0.9-2.0 | 0.908 (0.817, 1.009) | 0.072 | **0.875 (0.777, 0.986)** | **0.029** |  |  |
|  | >2.0 | **0.636 (0.492, 0.821)** | **<0.001** | **0.770 (0.630, 0.940)** | **0.010** |  |  |
|  |  |  |  |  |  |  |  |
|  | **TC, per unit** | 0.941 (0.860, 1.029) | 0.180 | **0.879 (0.810, 0.954)** | **0.002** | 0.891 (0.775, 1.023) | 0.101 |
|  | **TG, per unit** | **1.033 (1.008, 1.059)** | **0.011** | **1.042 (1.017, 1.067)** | **<0.001** | 0.973 (0.860, 1.100) | 0.661 |
|  | **LDLC, per unit** | 0.977 (0.891, 1.071) | 0.615 | 1.038 (0.932, 1.156) | 0.496 |  |  |
|  | **HDLC, per unit** | **0.776 (0.673, 0.895)** | **<0.001** | 0.958 (0.857, 1.070) | 0.447 |  |  |
| **Female** | **TC, mmol/L** |  |  |  |  |  |  |
|  | <3.1 | Ref |  | Ref |  |  |  |
|  | 3.1-5.7 | 0.779 (0.575, 1.055) | 0.106 | 0.897 (0.637, 1.264) | 0.536 | Ref |  |
|  | >5.7 | 0.777 (0.566, 1.067) | 0.118 | 0.822 (0.575, 1.175) | 0.282 | 0.888 (0.677, 1.166) | 0.394 |
|  | **TG, mmol/L** |  |  |  |  |  |  |
|  | <0.4 | Ref |  | Ref |  |  |  |
|  | 0.4-1.7 | 0.808 (0.527, 1.240) | 0.330 | 1.814 (0.572, 5.750) | 0.312 | Ref |  |
|  | >1.7 | 0.997 (0.646, 1.540) | 0.990 | 2.211 (0.696, 7.025) | 0.179 | 0.897 (0.645, 1.246) | 0.516 |
|  | **LDL-C, mmol/L** |  |  |  |  |  |  |
|  | <2.07 | Ref |  | Ref |  |  |  |
|  | 2.07-3.1 | 0.956 (0.865, 1.055) | 0.371 | **0.917 (0.844, 0.997)** | **0.041** |  |  |
|  | >3.1 | 0.951 (0.848, 1.067) | 0.400 | 0.939 (0.828, 1.065) | 0.325 |  |  |
|  | **HDL-C, mmol/L** |  |  |  |  |  |  |
|  | <0.9 | Ref |  | Ref |  |  |  |
|  | 0.9-2.0 | 0.864 (0.688, 1.084) | 0.206 | 1.024 (0.791, 1.326) | 0.856 |  |  |
|  | >2.0 | **0.708 (0.548, 0.915)** | **0.008** | 0.772 (0.576, 1.035) | 0.084 |  |  |
|  |  |  |  |  |  |  |  |
|  | **TC, per unit** | **0.786 (0.693, 0.891)** | **<0.001** | **0.874 (0.797, 0.958)** | **0.004** | 1.003 (0.882, 1.141) | 0.967 |
|  | **TG, per unit** | **1.126 (1.082, 1.172)** | **<0.001** | **1.089 (1.054, 1.125)** | **<0.001** | 0.925 (0.765, 1.119) | 0.423 |
|  | **LDLC, per unit** | **1.276 (1.122, 1.451)** | **<0.001** | **1.132 (1.002, 1.279)** | **0.047** |  |  |
|  | **HDLC, per unit** | 1.022 (0.868, 1.203) | 0.793 | 0.930 (0.818, 1.058) | 0.269 |  |  |

The ORs were adjusted for age, sex, BMI, fatty liver disease, kidney stone, hypertension, FBG, Cr, UA, UN, TBIL, ALT, AST, and TC, TG, LDL-C, LDL-C. Bold means *p* < 0.05, TC: total cholesterol, TG: triglycerides, LDL: low density lipoprotein cholesterol, HDL: high density lipoprotein cholesterol.
