# Supplementary material for: Blood lipid metabolism and the risk of gallstone disease: a multi-center study and meta-analysis
Source: Lipids Health Dis. 2022 Mar 2;21:26. doi: 10.1186/s12944-022-01635-9 (PMC8889751; doi:10.1186/s12944-022-01635-9)
Supplement: Supplementary file 5 — Additional file 5. The characteristics of the included publications regarding the mean difference of the blood lipid levels between groups. [file 12944_2022_1635_MOESM5_ESM.docx]

**Additional file 5.** The characteristics of the included publications regarding the mean difference of the blood lipid levels between groups

| **Study** | **Period** | **Study_design** | **Geographic background** | **Cases** | **Controls** | **Sex** | **Traits** | **GSD** | **Non_GSD** | **Units** |
| --- | --- | --- | --- | --- | --- | --- | --- | --- | --- | --- |
|  |  |  |  |  |  |  |  | **mean±SD** | **mean±SD** |  |
| Sepehrimanesh, 2020[1] | Jan. 2012 - Jan. 2018 | cross-sectional study | Asia | 59 | 177 | Both | TG | 137.2±103.8 | 124.2±107.9 | mg/dL |
|  |  |  |  |  |  | Both | HDL-C | 44.5±10.7 | 47.2±11.8 | mg/dL |
| Wang, 2020[2] | Jan. 2014 - Jan. 2015 | cross-sectional study | Asia | 168,092 | 1,900,432 | Both | TC | 5.0±1.0 | 4.8±1.0 | mmol/L |
|  |  |  |  |  |  | Both | TG | 1.7±1.3 | 1.5±1.3 | mmol/L |
|  |  |  |  |  |  | Both | LDL-C | 3.0±0.8 | 2.9±0.8 | mmol/L |
|  |  |  |  |  |  | Both | HDL-C | 1.3±0.4 | 1.3±0.3 | mmol/L |
| Song, 2020[3] | NA | cross-sectional study | Europe | 274 | 3,735 | Both | TC | 5.2±0.9 | 5.1±1.0 | mmol/L |
|  |  |  |  |  |  | Both | TG | 1.7±1.4 | 1.5±1.3 | mmol/L |
|  |  |  |  |  |  | Both | LDL-C | 3.3±0.8 | 3.2±0.9 | mmol/L |
|  |  |  |  |  |  | Both | HDL-C | 1.5±0.4 | 1.5±0.4 | mmol/L |
| Gu, 2020[4] | Jul. 2010 - Dec. 2012 | case-control study | Asia | 94 | 2,194 | Both | TC | 4.1±1.5 | 4.2±1.6 | mmol/L |
|  |  |  |  |  |  | Both | TG | 1.4±0.9 | 1.8±1.7 | mmol/L |
|  |  |  |  |  |  | Both | LDL-C | 3.5±1.9 | 2.7±1.0 | mmol/L |
|  |  |  |  |  |  | Both | HDL-C | 1.8±0.9 | 1.8±1.2 | mmol/L |
| Kim, 2019[5] | Jan. 2009 - Dec. 2017 | cross-sectional study | Asia | 355 | 7,531 | Both | TC | 198.6±36.7 | 198.9±37.2 | mg/dL |
|  |  |  |  |  |  | Both | TG | 121.8±76.0 | 118.8±91.0 | mg/dL |
|  |  |  |  |  |  | Both | LDL-C | 122.3±33.8 | 121.1±34.3 | mg/dL |
|  |  |  |  |  |  | Both | HDL-C | 52.0±13.6 | 54.1±13.7 | mg/dL |
| Hayat, 2019[6] | Aug. 2017 - Aug. 2018 | cross-sectional study | Asia | 50 | 50 | Both | TC | 184.6±37.7 | 181.1±34.0 | mg/dL |
|  |  |  |  |  |  | Both | TG | 198.1±48.4 | 172.0±54.6 | mg/dL |
|  |  |  |  |  |  | Both | LDL-C | 118.4±24.0 | 122.1±35.9 | mg/dL |
|  |  |  |  |  |  | Both | HDL-C | 29.5±8.4 | 40.3±11.6 | mg/dL |
| Kim, 2019[7] | Jun. 2014 - May. 2015 | cross-sectional study | Asia | 806 | 36,495 | Male | TC | 110.8±27.1 | 110.3±27.5 | mg/dL |
|  |  |  | Asia | 554 | 21,544 | Female | TC | 97.9±24.6 | 94.9±24.2 | mg/dL |
|  |  |  | Europe | 806 | 36,495 | Male | LDL-C | 189.3±30.9 | 190.5±30.2 | mg/dL |
|  |  |  | Europe | 554 | 21,544 | Female | LDL-C | 178.9±28.2 | 177.7±26.8 | mg/dL |
|  |  |  | Asia | 806 | 36,495 | Male | HDL-C | 53.2±11.6 | 55.0±12.4 | mg/dL |
|  |  |  | Asia | 554 | 21,544 | Female | HDL-C | 65.1±13.9 | 68.4±13.7 | mg/dL |
| Dhamnetiya, 2018[8] | Jan. 2013 - Dec. 2013 | case-control study | Asia | 120 | 120 | Both | TC | 183.5±34.4 | 163.7±27.8 | mg/dL |
|  |  |  |  |  |  | Both | TG | 169.1±63.2 | 149.8±19.4 | mg/dL |
|  |  |  |  |  |  | Both | LDL-C | 123.9±19.7 | 113±18.8 | mg/dL |
|  |  |  |  |  |  | Both | HDL-C | 40.9±8.1 | 41.3±7.8 | mg/dL |
| Kwon, 2018[9] | Jan. 2003 - Dec. 2015 | cross-sectional study | Asia | 821 | 19,942 | Both | TC | 193.9±36.7 | 196.3±37.2 | mg/dL |
|  |  |  |  |  |  | Both | TG | 118.2±75.7 | 117.4±85 | mg/dL |
|  |  |  |  |  |  | Both | LDL-C | 118.7±33.4 | 118.7±33.7 | mg/dL |
|  |  |  |  |  |  | Both | HDL-C | 52.2±13.3 | 54.5±13.6 | mg/dL |
| Serin, 2017[10] | Jan. 2015 - Mar. 2015 | cross-sectional study | Other | 48 | 88 | Both | TC | 184.9±45.1 | 205.2±50.3 | mg/dL |
| Ravikanth, 2016[11] | NA | case-control study | Asia | 305 | 177 | Both | TC | 171.0±36.7 | 169.0±24.1 | mg/dL |
|  |  |  |  |  |  | Both | TG | 148.0±70.0 | 152.0±76.6 | mg/dL |
|  |  |  |  |  |  | Both | LDL-C | 104.0±31.8 | 100.0±16.1 | mg/dL |
|  |  |  |  |  |  | Both | HDL-C | 37.6±16.9 | 39.4±25.8 | mg/dL |
| Zhan, 2016[12] | NA | cross-sectional study | Asia | 171 | 125 | Both | LDL-C | 130.7±35.1 | 113.3±28.9 | mg/dL |
|  |  |  |  |  |  | Both | HDL-C | 46.8±10.5 | 51.8±12.0 | mg/dL |
| Zhang, 2015[13] | Jan. 2010 - Jan. 2014 | cross-sectional study | Asia | 882 | 9,134 | Both | TG | 1.8±3.9 | 1.5±1.3 | mg/dL |
|  |  |  |  |  |  | Both | TC | 4.9±1.1 | 4.7±1.0 | mmol/L |
|  |  |  |  |  |  | Both | LDL-C | 2.9±6.7 | 2.6±2.0 | mmol/L |
|  |  |  |  |  |  | Both | HDL-C | 1.2±0.4 | 1.3±2.4 | mmol/L |
| Dai, 2015[14] | Jun. 2012 - Oct. 2013 | case-control study | Asia | 20 | 10 | Both | TC | 3.9±1.3 | 3.2±0.6 | mmol/L |
|  |  |  |  |  |  | Both | TG | 1.3±0.2 | 1.2±0.2 | mmol/L |
| Dwivedi, 2015[15] | NA | case-control study | Asia | 102 | 256 | Both | TC | 175.4±67.4 | 156.7±38.0 | mg/dL |
|  |  |  |  |  |  | Both | TG | 165.5±63.3 | 125.8±59.6 | mg/dL |
|  |  |  |  |  |  | Both | LDL-C | 113.3±68.9 | 90.4±39.7 | mg/dL |
|  |  |  |  |  |  | Both | HDL-C | 29.0±13.3 | 41.1±12.2 | mg/dL |
| Kwak, 2015[16] | Jan. 2010 - Dec. 2010 | cross-sectional study | Asia | 1,069 | 16,543 | Both | TC | 194.4±33.7 | 193.4±33.7 | mg/dL |
|  |  |  |  |  |  | Both | TG | 103.2±68.0 | 111.1±62.3 | mg/dL |
|  |  |  |  |  |  | Both | HDL-C | 55.1±12.2 | 53.2±12.1 | mg/dL |
| Martinez-Lopez, 2015[17] | Apr. 2007 - Dec. 2009 | case-control study | America | 90 | 371 | Both | TC | 172.9±41.5 | 184.9±36.3 | mg/dL |
|  |  |  |  |  |  | Both | TG | 146.6±60.2 | 145±87.5 | mg/dL |
|  |  |  |  |  |  | Both | LDL-C | 128.2±10.0 | 110.9±30.9 | mg/dL |
|  |  |  |  |  |  | Both | HDL-C | 40.3±7.7 | 45.0±19.2 | mg/dL |
| Sarac, 2015[18] | Nov. 2008 - Dec. 2010 | case-control study | Other | 90 | 50 | Both | TC | 190.7±27.8 | 162.4±11.4 | mg/dL |
|  |  |  |  |  |  | Both | TG | 160±20.4 | 112.3±10.9 | mg/dL |
|  |  |  |  |  |  | Both | HDL-C | 32.1±14.9 | 45.2±12.4 | mg/dL |
| Zamani, 2014[19] | 2008 – 2010 | cross-sectional study | Asia | 51 | 6,143 | Both | TC | 194.6±42.1 | 180.3±47.4 | mg/dL |
|  |  |  |  |  |  | Both | TG | 166.9±131.8 | 134±110.2 | mg/dL |
| Zhu, 2014[20] | Mar. 2013 - Jun. 2013 | cross-sectional study | Asia | 1,240 | 8,215 | Both | TC | 4.9±0.9 | 4.8±1.0 | mmol/L |
|  |  |  |  |  |  | Both | TG | 2.0±1.4 | 1.8±1.5 | mmol/L |
|  |  |  |  |  |  | Both | LDL-C | 2.8±0.8 | 2.8±1.6 | mmol/L |
|  |  |  |  |  |  | Both | HDL-C | 1.3±0.3 | 1.3±0.4 | mmol/L |
| Lin, 2014[21] | 2011 - 2012 | cross-sectional study | Asia | 734 | 11,180 | Both | TC | 191.8±37.3 | 192.8±35.7 | mg/dL |
|  |  |  |  |  |  | Both | TG | 126.8±111.9 | 115.1±105 | mg/dL |
|  |  |  |  |  |  | Both | LDL-C | 119.9±33.2 | 119.9±31 | mg/dL |
|  |  |  |  |  |  | Both | HDL-C | 46.1±12.1 | 49.2±12.8 | mg/dL |
| Lee, 2014[22] | Jan. 2000 - Aug. 2009 | cross-sectional study | Asia | 768 | 11,265 | Both | TC | 202.2±38.4 | 197.6±36.8 | mg/dL |
|  |  |  |  |  |  | Both | TG | 140.0±83.1 | 132.0±88.5 | mg/dL |
|  |  |  |  |  |  | Both | HDL-C | 47.7±12.5 | 49.3±13.7 | mg/dL |
| Ajdarkosh, 2013[23] | Jan. 2008 - Feb. 2012 | case-control study | Asia | 151 | 347 | Both | TC | 189.8±61.1 | 188.2±43.1 | mg/dL |
|  |  |  |  |  |  | Both | TG | 169.8±133 | 143.1±101 | mg/dL |
|  |  |  |  |  |  | Both | LDL-C | 64.8±39.5 | 111.0±39.7 | mg/dL |
|  |  |  |  |  |  | Both | HDL-C | 73.4±43.1 | 46.4±13.6 | mg/dL |
| Chen, 2014[24] | 2002 - Dec. 2007 | cohort study | Asia | 23 | 1,273 | Both | TC | 218.2±35.7 | 209.8±36.8 | mg/dL |
|  |  |  |  |  |  | Both | TG | 150.7±88.0 | 147.3±79.5 | mg/dL |
|  |  |  |  |  |  | Both | HDL-C | 55.7±16.3 | 54.2±15.9 | mg/dL |
| Batajoo, 2013[25] | Jan. 2010 - Dec. 2011 | cross-sectional study | Asia | 61 | 67 | Female | TC | 189.3±34.0 | 178.6±28.6 | mg/dL |
|  |  |  |  |  |  | Female | TG | 130.4±48.5 | 125.2±42.2 | mg/dL |
|  |  |  |  |  |  | Female | LDL-C | 113.5±32.7 | 102.0±27.9 | mg/dL |
|  |  |  |  |  |  | Female | HDL-C | 42.2±3.4 | 43.1±2.4 | mg/dL |
| Takahashi, 2014[26] | 2010 | cross-sectional study | Asia | 694 | 14,857 | Both | TC | 205.4±33.3 | 201.6±32.6 | mg/dL |
|  |  |  |  |  |  | Both | TG | 121.2±121.2 | 103.1±72.2 | mg/dL |
|  |  |  |  |  |  | Both | LDL-C | 127.6±30.7 | 123.2±31.0 | mg/dL |
|  |  |  |  |  |  | Both | HDL-C | 62.3±16.9 | 65.4±16.8 | mg/dL |
| Chen, 2012[27] | NA | cross-sectional study | Asia | 918 | 6,652 | Both | TG | 201.1±183.3 | 183.7±182.7 | mg/L |
|  |  |  |  |  |  | Both | HDL-C | 44.6±10.9 | 46.0±0.6 | mg/L |
| Kim, 2011[28] | Jan. 2006 - Dec. 2007 | cross-sectional study | Asia | 173 | 3,952 | Female | TC | 200.7±35.6 | 196.3±34.7 | mg/dL |
|  |  |  |  |  |  | Female | TG | 103.0±51.7 | 98.0±55.7 | mg/dL |
|  |  |  |  |  |  | Female | LDL-C | 124.8±31.3 | 120.2±31.0 | mg/dL |
|  |  |  |  |  |  | Female | HDL-C | 58.8±13.9 | 60.5±13.8 | mg/dL |
| Karayalcin, 2010[29] | May. 2007 - Nov. 2007 | cross-sectional study | Other | 73 | 401 | Female | TC | 216.5±44.9 | 215.9±44.4 | mg/dL |
|  |  |  |  |  |  | Female | TG | 134.5±54.8 | 143.3±77.1 | mg/dL |
|  |  |  |  |  |  | Female | LDL-C | 138.3±38.4 | 130.7±40.7 | mg/dL |
|  |  |  |  |  |  | Female | HDL-C | 51.7±14.0 | 55.7±19.3 | mg/dL |
| Wang, 2010[30] | Jan. 2008 - Jul. 2008 | case-control study | Asia | 100 | 147 | Both | TC | 199.0±50.4 | 181.4±33.8 | mg/dL |
|  |  |  |  |  |  | Both | TG | 140.6±81.1 | 104.0±56.8 | mg/dL |
| Siddapuram, 2010[31] | NA | case-control study | Asia | 226 | 289 | Both | TC | 182.6±56.0 | 225.0±0.4 | mg/dL |
|  |  |  |  |  |  | Both | TG | 149.7±84.0 | 147.2±89.2 | mg/dL |
|  |  |  |  |  |  | Both | LDL-C | 120.3±43.3 | 138.9±39.7 | mg/dL |
|  |  |  |  |  |  | Both | HDL-C | 32.2±9.1 | 39.1±9.8 | mg/dL |
| Tirziu, 2008[32] | Nov. 2002 - Sep. 2007 | case-control study | Europe | 109 | 271 | Both | TC | 213.4±49.9 | 226.5±48.4 | mg/dL |
|  |  |  |  |  |  | Both | TG | 172.9±95.9 | 133.4±78.4 | mg/dL |
|  |  |  |  |  |  | Both | HDL-C | 48.8±14.9 | 55.4±13.9 | mg/dL |
| Chang, 2008[33] | Jan. 2005 - Oct. 2005 | cross-sectional study | Asia | 440 | 19,063 | Male | TC | 196.5±34.3 | 195.8±32.3 | mg/dL |
|  |  |  |  |  |  | Male | TG | 145.1±83.9 | 148.5±89.3 | mg/dL |
|  |  |  |  |  |  | Male | LDL-C | 115.8±28.4 | 114.7±27.2 | mg/dL |
|  |  |  |  |  |  | Male | HDL-C | 50.0±11.0 | 50.4±10.3 | mg/dL |
| Kuo, 2008[34] | 2005 | cross-sectional study | Asia | 74 | 905 | Both | TC | 167.3±32.0 | 182.8±34 | mg/dL |
|  |  |  |  |  |  | Both | TG | 124.2±37.7 | 138±116.5 | mg/dL |
|  |  |  |  |  |  | Both | LDL-C | 110.4±28.5 | 123.8±33.5 | mg/dL |
|  |  |  |  |  |  | Both | HDL-C | 48.8±10.2 | 53.0±13.7 | mg/dL |
| Andreotti, 2008[35] | Jun. 1997 - May. 2001 | case-control study | Asia | 981 | 858 | Both | TC | 170.9±43.9 | 182.3±55.7 | mg/dL |
|  |  |  |  |  |  | Both | TG | 127.4±68.9 | 107.3±67.4 | mg/dL |
|  |  |  |  |  |  | Both | LDL-C | 101.5±43.9 | 108.2±52.7 | mg/dL |
|  |  |  |  |  |  | Both | HDL-C | 37.4±12.5 | 43.9±14.7 | mg/dL |
| Wang, 2007[36] | NA | case-control study | Asia | 287 | 205 | Both | TC | 4.3±1.2 | 4.1±1.3 | mmol/L |
|  |  |  |  |  |  | Both | TG | 1.7±1.0 | 1.7±1.9 | mmol/L |
|  |  |  |  |  |  | Both | LDL-C | 2.4±1.2 | 2.1±1.2 | mmol/L |
|  |  |  |  |  |  | Both | HDL-C | 1.2±0.5 | 1.2±0.4 | mmol/L |
| Mella, 2007[37] | NA | case-control study | America | 117 | 122 | Both | TC | 198.0±39.0 | 187.0±39.0 | mg/dL |
|  |  |  | Europe | 184 | 184 | Both | TC | 200.0±53.0 | 202.0±55.0 | mg/dL |
|  |  |  | America | 117 | 122 | Both | TG | 135.0±86.0 | 120.0±78.0 | mg/dL |
|  |  |  | Europe | 184 | 184 | Both | TG | 143.0±79.0 | 146.0±105.0 | mg/dL |
|  |  |  | America | 117 | 122 | Both | LDL-C | 124.0±36.0 | 119.0±3.0 | mg/dL |
|  |  |  | Europe | 184 | 184 | Both | LDL-C | 122.0±46.0 | 125.0±42.0 | mg/dL |
|  |  |  | America | 117 | 122 | Both | HDL-C | 47.0±11.0 | 44.0±11.0 | mg/dL |
|  |  |  | Europe | 184 | 184 | Both | HDL-C | 48.0±19.0 | 50.0±20.0 | mg/dL |
| Acalovschi, 2006[38] | NA | case-control study | Europe | 34 | 68 | Both | TC | 202.8±34.9 | 207.2±62.4 | mg/dL |
|  |  |  |  |  |  | Both | TG | 163.8±69.7 | 133.2±70.2 | mg/dL |
|  |  |  |  |  |  | Both | HDL-C | 40.3±11.6 | 58.9±16.4 | mg/dL |
| Méndez-Sánchez, 2006[39] | Jun. 2003 - Apr. 2004 | cross-sectional study | America | 54 | 43 | Both | TC | 208.4±44.5 | 203.6±34.2 | mg/dL |
|  |  |  |  |  |  | Both | TG | 186.5±139.6 | 171.4±97.5 | mg/dL |
|  |  |  |  |  |  | Both | LDL-C | 135.5±48.5 | 130.3±26.0 | mg/dL |
|  |  |  |  |  |  | Both | HDL-C | 40.3±10.8 | 39.6±11.3 | mg/dL |
| Liu, 2006[40] | Jan. 2002 - Dec. 2007 | cross-sectional study | Asia | 126 | 2,260 | Both | TC | 216.9±41.1 | 210.5±37.7 | mg/dL |
|  |  |  |  |  |  | Both | TG | 143.4±98.6 | 130.1±108.1 | mg/dL |
|  |  |  |  |  |  | Both | HDL-C | 57.0±16.6 | 57.7±15.8 | mg/dL |
| Nervi, 2006[41] | 1993 - 2000 | nested case-control study | America | 299 | 582 | Both | TC | 5.4±1.1 | 5.4±1.3 | mmol/L |
|  |  |  |  |  |  | Both | TG | 1.5±1.0 | 1.3±0.7 | mmol/L |
|  |  |  |  |  |  | Both | LDL-C | 3.4±0.9 | 3.4±1.0 | mmol/L |
|  |  |  |  |  |  | Both | HDL-C | 1.3±0.3 | 1.4±0.3 | mmol/L |
| Wang, 2006[42] | Feb. 2004 - Sep. 2004 | case-control study | Asia | 90 | 91 | Both | TC | 200.4±40.4 | 197.8±33.2 | mg/dL |
|  |  |  |  |  |  | Both | TG | 143.7±100.9 | 119.8±74.7 | mg/dL |
| Sakuta, 2005[43] | NA | cross-sectional study | Asia | 39 | 926 | Female | TC | 214.0±34.0 | 213.0±31.0 | mg/dL |
|  |  |  |  |  |  | Female | TG | 143.0±77.0 | 146.0±61.0 | mg/dL |
| Méndez-Sánchez, 2005[44] | NA | case-control study | America | 97 | 190 | Both | TC | 5.4±1.1 | 5.4±1.0 | mmol/L |
|  |  |  |  |  |  | Both | TG | 1.9±1.2 | 1.7±1.0 | mmol/L |
|  |  |  |  |  |  | Both | LDL-C | 3.4±0.9 | 3.5±0.9 | mmol/L |
|  |  |  |  |  |  | Both | HDL-C | 1.2±0.4 | 1.2±0.4 | mmol/L |
| Mendez-Sanchez, 2005[45] | NA | cross-sectional study | America | 65 | 180 | both | TC | 5.3±1.2 | 5.3±1.1 | mmol/L |
|  |  |  |  |  |  | both | HDL-C | 1.0±0.3 | 1.1±0.3 | mmol/L |
|  |  |  |  |  |  | both | LDL-C | 3.3±0.9 | 3.4±0.9 | mmol/L |
|  |  |  |  |  |  | both | TG | 1.9±0.9 | 1.8±1.2 | mmol/L |
| Volzke, 2005[46] | NA | cross-sectional study | Europe | 891 | 3,311 | Both | TC | 5.9±1.3 | 5.7±1.2 | mmol/L |
|  |  |  |  |  |  | Both | LDL-C | 3.7±1.2 | 3.6±1.2 | mmol/L |
|  |  |  |  |  |  | Both | HDL-C | 1.5±0.4 | 1.4±0.5 | mmol/L |
| Jiang, 2004[47] | Feb. 1998 - May. 1998 | case-control study | Asia | 105 | 274 | Both | TC | 4.7±0.9 | 4.9±1.0 | mmol/L |
|  |  |  |  |  |  | Both | TG | 1.3±1.2 | 1.2±0.8 | mmol/L |
|  |  |  |  |  |  | Both | LDL-C | 2.6±0.7 | 2.7±0.7 | mmol/L |
|  |  |  |  |  |  | Both | HDL-C | 1.3±0.4 | 1.4±0.3 | mmol/L |
| Galman, 2004[48] | 1992 - 2000 | cross-sectional study | America | 45 | 80 | Female | TC | 5.4±5.9 | 1.3±1.0 | mmol/L |
|  |  |  | America | 20 | 20 | Female | TC | 4.8±1.1 | 4.7±1.2 | mmol/L |
|  |  |  | America | 45 | 80 | Female | TG | 1.6±1.5 | 0.7±0.7 | mmol/L |
|  |  |  | America | 20 | 20 | Female | TG | 1.6±1.1 | 1.3±0.5 | mmol/L |
|  |  |  | America | 45 | 80 | Female | LDL-C | 3.4±3.8 | 1.1±0.9 | mmol/L |
|  |  |  | America | 20 | 20 | Female | LDL-C | 2.9±1.0 | 2.9±0.9 | mmol/L |
|  |  |  | America | 45 | 80 | Female | HDL-C | 1.3±1.4 | 0.3±0.3 | mmol/L |
|  |  |  | America | 20 | 20 | Female | HDL-C | 1.2±0.4 | 1.1±0.3 | mmol/L |
| Hasegawa, 2003[49] | NA | case-control study | Asia | 79 | 53 | Both | TC | 159.0±44.4 | 173.0±29.1 | mg/dL |
|  |  |  |  |  |  | Both | TG | 81.0±44.4 | 97.0±43.7 | mg/dL |
|  |  |  |  |  |  | Both | LDL-C | 100.0±35.6 | 106.0±29.1 | mg/dL |
|  |  |  |  |  |  | Both | HDL-C | 41.0±8.9 | 45.0±7.3 | mg/dL |
| Gustafsson, 2003[50] | NA | case-control study | Europe | 81 | 36 | Both | TC | 5.7±0.9 | 5.8±1.2 | mmol/L |
|  |  |  |  |  |  | Both | TG | 1.5±0.9 | 1.4±0.6 | mmol/L |
| Kurtul, 2002[51] | NA | case-control study | Other | 32 | 32 | Both | TC | 209.2±11.6 | 185.6±19.3 | mg/dL |
|  |  |  |  |  |  | Both | TG | 186.9±9.4 | 136.4±23.2 | mg/dL |
|  |  |  |  |  |  | Both | LDL-C | 132.7±8.0 | 95.4±13.7 | mg/dL |
|  |  |  |  |  |  | Both | HDL-C | 43.4±3.6 | 48.4±7.8 | mg/dL |
| Devesa, 2001[52] | Feb. 1991 - Apr. 1993 | cross-sectional study | Asia | 92 | 572 | Female | TC | 244.5±58.0 | 220.7±48.4 | mg/dL |
|  |  |  | Asia | 34 | 564 | Male | TC | 224.4±44.1 | 223.1±48.4 | mg/dL |
|  |  |  | Asia | 92 | 572 | Female | TG | 123.4±80.0 | 90.6±63.1 | mg/dL |
|  |  |  | Asia | 34 | 564 | Male | TG | 151.9±131.9 | 133.2±90.2 | mg/dL |
|  |  |  | Europe | 126 | 1,136 | Both | LDL-C | 151.3±49.5 | 141.8±41.7 | mg/dL |
|  |  |  | Europe | 126 | 1,136 | Both | HDL-C | 62.1±15.2 | 60.6±16.6 | mg/dL |
| Misciagna, 2000[53] | May. 1985 - Jun. 1993 | case-control study | Europe | 54 | 162 | Male | TC | 5.0±1.0 | 5.2±1.1 | mmol/L |
|  |  |  | Asia | 47 | 141 | Female | TC | 5.5±1.3 | 5.1±1.1 | mmol/L |
|  |  |  | Asia | 54 | 162 | Male | TG | 1.5±0.7 | 1.6±1.3 | mmol/L |
|  |  |  | Asia | 47 | 141 | Female | TG | 1.7±1.3 | 1.3±0.9 | mmol/L |
|  |  |  | Europe | 54 | 162 | Male | HDL-C | 1.2±0.3 | 1.2±0.3 | mmol/L |
|  |  |  | Europe | 47 | 141 | Female | HDL-C | 1.3±0.3 | 1.3±0.3 | mmol/L |
| Han, 2000[54] | Jan. 1998 - May. 1998 | case-control study | Asia | 190 | 441 | Both | TC | 4.6±0.9 | 4.8±1.0 | mmol/L |
|  |  |  |  |  |  | Both | TG | 1.4±1.1 | 1.2±0.9 | mmol/L |
|  |  |  |  |  |  | Both | LDL-C | 2.6±0.7 | 2.6±0.7 | mmol/L |
|  |  |  |  |  |  | Both | HDL-C | 1.3±0.3 | 1.4±0.3 | mmol/L |
| Chen, 1999[55] | NA | case-control study | Asia | 236 | 1,092 | Both | TC | 192.5±36.1 | 192.9±38.1 | mg/dL |
|  |  |  |  |  |  | Both | TG | 160.4±128.8 | 138.8±92.9 | mg/dL |
|  |  |  |  |  |  | Both | LDL-C | 119.9±30.4 | 121.1±31.7 | mg/dL |
|  |  |  |  |  |  | Both | HDL-C | 42.8±12.2 | 45.5±14.0 | mg/dL |
| Duque, 1999[56] | Aug. 1991 - Aug. 1992 | cross-sectional study | America | 113 | 1,463 | Male | TC | 209.9±46.9 | 214.9±44.6 | mg/dL |
|  |  |  |  | 72 | 441 | Female | TC | 205.5±45.0 | 205.1±48.7 | mg/dL |
|  |  |  |  | 185 | 1,904 | Both | TG | 5.0±0.6 | 5.0±0.6 | mg/dL |
|  |  |  |  | 113 | 1,463 | Male | HDL-C | 42.8±13.4 | 42.2±11.9 | mg/dL |
|  |  |  |  | 72 | 441 | Female | HDL-C | 50.9±14.3 | 50.4±12.5 | mg/dL |
| Sasazuki, 1999[57] | Oct. 1986 - Dec. 1994 | cross-sectional study | Asia | 277 | 6,895 | Male | TG | 4.8±0.5 | 4.7±0.8 | mg/dL |
| Niemi, 1999[58] | NA | cross-sectional study | Europe | 47 | 220 | Female | TC | 5.7±1.1 | 5.5±0.8 | mmol/L |
|  |  |  |  | 17 | 242 | Male | TC | 5.1±0.6 | 5.8±0.8 | mmol/L |
|  |  |  |  | 47 | 220 | Female | TG | 1.5±1.1 | 1.1±0.8 | mmol/L |
|  |  |  |  | 17 | 242 | Male | TG | 1.6±0.8 | 1.6±0.8 | mmol/L |
|  |  |  |  | 47 | 220 | Female | LDL-C | 3.4±1.1 | 3.3±0.8 | mmol/L |
|  |  |  |  | 17 | 242 | Male | LDL-C | 3.2±0.8 | 3.8±1.6 | mmol/L |
|  |  |  |  | 47 | 220 | Female | HDL-C | 1.5±0.4 | 1.6±0.8 | mmol/L |
|  |  |  |  | 17 | 242 | Male | HDL-C | 1.1±0.2 | 1.2±0.8 | mmol/L |
| Chen, 1998[59] | Jan. 1995 - Jul. 1995 | cross-sectional study | Asia | 386 | 2,946 | Both | TC | 4.9±1.0 | 4.9±0.9 | mmol/L |
|  |  |  |  |  |  | Both | TG | 1.7±1.4 | 1.8±1.4 | mmol/L |
|  |  |  |  |  |  | Both | LDL-C | 3.0±0.8 | 3.1±0.8 | mmol/L |
|  |  |  |  |  |  | Both | HDL-C | 1.2±0.4 | 1.1±0.3 | mmol/L |
| Borch, 1998[60] | NA | cross-sectional study | Europe | 59 | 57 | Female | TC | 6.6±1.2 | 6.6±1.2 | mmol/L |
|  |  |  |  | 45 | 44 | Male | TC | 6.1±1.1 | 6.4±1.2 | mmol/L |
|  |  |  |  | 58 | 57 | Female | TG | 1.7±1.0 | 1.6±1.0 | mmol/L |
|  |  |  |  | 45 | 44 | Male | TG | 1.9±0.9 | 1.6±0.8 | mmol/L |
|  |  |  |  | 45 | 41 | Female | LDL-C | 4.1±1.1 | 4.1±1.0 | mmol/L |
|  |  |  |  | 24 | 24 | Male | LDL-C | 3.9±0.9 | 4.0±1.0 | mmol/L |
|  |  |  |  | 51 | 45 | Female | HDL-C | 1.5±0.4 | 1.6±0.5 | mmol/L |
|  |  |  |  | 28 | 28 | Male | HDL-C | 1.1±0.3 | 1.3±0.4 | mmol/L |
| Fu, 1997[61] | Jan. 1994 - Jun. 1994 | case-control study | Asia | 47 | 19 | Both | TC | 4.2±1.1 | 4.5±0.8 | mmol/L |
|  |  |  |  | 47 | 19 | Both | TG | 1.5±0.6 | 1.2±0.4 | mmol/L |
|  |  |  |  | 47 | 19 | Both | LDL-C | 1.8±1.0 | 2.3±1.1 | mmol/L |
|  |  |  |  | 47 | 19 | Both | HDL-C | 1.1±0.6 | 1.2±0.3 | mmol/L |
| Miquel, 1998[62] | NA | cross-sectional study | America | 52 | 40 | Both | TC | 181.0±10.0 | 205.0±7.0 | mg/dL |
| Singh, 1997[63] | NA | cross-sectional study | Asia | 50 | 26 | Both | TC | 262.9±39.2 | 224.7±29.3 | mg/dL |
|  |  |  |  |  |  | Both | TG | 157±25.7 | 124.1±24.7 | mg/dL |
|  |  |  |  |  |  | Both | LDL-C | 180.8±24.9 | 121.8±22.4 | mg/dL |
|  |  |  |  |  |  | Both | HDL-C | 42.7±10.0 | 65.3±12.5 | mg/dL |
| Tang, 1996[64] | NA | cross-sectional study | Asia | 51 | 19 | Both | TC | 138.2±28.9 | 144.7±35.2 | mg/dL |
|  |  |  |  |  |  | Both | TG | 101.7±50.0 | 91.1±30.2 | mg/dL |
|  |  |  |  |  |  | Both | LDL-C | 99.9±25.5 | 113.5±29.1 | mg/dL |
|  |  |  |  |  |  | Both | HDL-C | 26.4±7.1 | 23.1±4.6 | mg/dL |
| Bertomeu, 1996[65] | Apr. 1992 - May. 1994 | case-control study | Europe | 160 | 125 | Both | TC | 243.0±49.3 | 228.0±45.8 | mg/dL |
|  |  |  |  |  |  | Both | TG | 133.0±73.4 | 126.0±79.4 | mg/dL |
|  |  |  |  |  |  | Both | LDL-C | 168.0±45.5 | 156.0±51.4 | mg/dL |
|  |  |  |  |  |  | Both | HDL-C | 50.0±12.7 | 53.0±16.8 | mg/dL |
| Villalpando, 1997[66] | NA | cross-sectional study | America | 124 | 1,211 | Female | TC | 196.6±43.6 | 190.9±44.2 | mg/dL |
|  |  |  |  | 19 | 920 | Male | TC | 178.6±35.8 | 191.8±41.9 | mg/dL |
|  |  |  |  | 19 | 920 | Male | TG | 226.3±103.6 | 246.4±175.5 | mg/dL |
|  |  |  |  | 124 | 1,211 | Female | TG | 195.2±93.5 | 185.3±123.6 | mg/dL |
|  |  |  |  | 19 | 920 | Male | LDL-C | 117.1±33.3 | 123.4±38.0 | mg/dL |
|  |  |  |  | 124 | 1,211 | Female | LDL-C | 126.1±36.8 | 122.6±40.4 | mg/dL |
|  |  |  |  | 19 | 920 | Male | HDL-C | 26.4±4.1 | 30.3±8.5 | mg/dL |
|  |  |  |  | 124 | 1,211 | Female | HDL-C | 34.5±8.5 | 34.8±9.0 | mg/dL |
| Shinchi, 1993[67] | Oct. 1986 - Dec. 1990 | case-control study | Asia | 61 | 2,494 | Male | TC | 189.9±30.5 | 195.3±35 | mg/dL |
|  |  |  |  |  |  | Male | TG | 4.8±0.6 | 4.7±0.5 | mg/dL |
|  |  |  |  |  |  | Male | LDL-C | 109.7±32.8 | 116.5±35.0 | mg/dL |
|  |  |  |  |  |  | Male | HDL-C | 53.2±14.1 | 53.7±15.0 | mg/dL |
| Loria, 1994[68] | Nov. 1985 - Apr. 1986 | cross-sectional study | Europe | 26 | 967 | Male | TC | 205.8±6.8 | 210.8±251.0 | mg/100 ml |
|  |  |  |  | 35 | 837 | Female | TC | 203.5±7.2 | 197.1±167.2 | mg/100 ml |
|  |  |  |  | 26 | 967 | Male | TG | 2.1±0.1 | 2.1±1.6 | mg/100 ml |
|  |  |  |  | 35 | 837 | Female | TG | 1.9±0.1 | 2.0±0.9 | mg/100 ml |
|  |  |  |  | 26 | 967 | Male | HDL-C | 49.1±1.8 | 52.2±67.8 | mg/100 ml |
|  |  |  |  | 35 | 837 | Female | HDL-C | 58.1±2.7 | 53.5±62.2 | mg/100 ml |
| Juvonen, 1995[69] | Aug. 1989 - Feb. 1990 | case-control study | Europe | 93 | 92 | Both | TC | 5.3±1.2 | 5.7±1.2 | mmol/L |
|  |  |  |  | 93 | 92 | Both | TG | 1.4±0.7 | 1.3±0.6 | mmol/L |
|  |  |  |  | 93 | 92 | Both | LDL-C | 3.0±1.0 | 3.4±2.4 | mmol/L |
|  |  |  |  | 93 | 92 | Both | HDL-C | 1.3±0.3 | 1.4±0.4 | mmol/L |
| Sarin, 1995[70] | NA | cross-sectional study | Asia | 105 | 105 | Both | TC | 197.0±11.0 | 181±13.0 | mg/dL |
|  |  |  |  | 105 | 105 | Both | TG | 129.0±14.0 | 125±9.0 | mg/dL |
| Scragg, 1984[71] | Dec. 1978 - Sep. 1980 | case-control study | Oceania | 127 | 182 | Female | TC | 5.5±0.1 | 5.6±0.1 | mmol/L |
|  |  |  |  | 46 | 102 | Male | TC | 5.4±0.2 | 5.4±0.1 | mmol/L |
|  |  |  |  | 124 | 175 | Female | TG | 1.4±0.1 | 1.2±0.1 | mmol/L |
|  |  |  |  | 44 | 99 | Male | TG | 1.7±0.2 | 1.5±0.1 | mmol/L |
|  |  |  |  | 127 | 182 | Female | HDL-C | 1.2±0.02 | 1.2±0.02 | mmol/L |
|  |  |  |  | 46 | 102 | Male | HDL-C | 1.0±0.03 | 1.1±0.02 | mmol/L |
| GREPCO, 1988[72] | NA | cross-sectional study | Europe | 65 | 1,137 | Male | TC | 217.3±40.3 | 209.5±43.8 | mg/dL |
|  |  |  |  | 66 | 979 | Female | TC | 205.9±39.9 | 202.8±37.0 | mg/dL |
|  |  |  |  | 65 | 1,137 | Male | LDL-C | 144.8±37.2 | 133.5±47.4 | mg/dL |
|  |  |  |  | 66 | 979 | Female | LDL-C | 132.1±36.1 | 128.6±41.5 | mg/dL |
|  |  |  |  | 65 | 1,137 | Male | HDL-C | 44.0±11.2 | 39.2±12.1 | mg/dL |
|  |  |  |  | 66 | 979 | Female | HDL-C | 56.6±11.0 | 53.1±14.7 | mg/dL |
|  |  |  |  | 65 | 1,137 | Male | TG | 4.8±0.7 | 5.0±0.8 | mmol/L |
|  |  |  |  | 66 | 979 | Female | TG | 4.4±0.3 | 4.5±0.6 | mmol/L |
| Mellstrom, 1988[73] | 1906 - 1907 | cross-sectional study | Europe | 29 | 54 | Female | TC | 6.3±1.0 | 6.4±1.3 | mmol/L |
|  |  |  |  | 29 | 54 | Female | TG | 1.7±0.8 | 1.3±0.7 | mmol/L |
| Mohr, 1991[74] | 1984 - 1987 | cross-sectional study | America | 214 | 1,089 | Female | TC | 225.0±39.4 | 229.6±39.1 | mg/dL |
|  |  |  |  | 214 | 1,089 | Female | TG | 122.5±1.7 | 98.0±1.7 | mg/dL |
|  |  |  |  | 214 | 1,089 | Female | LDL-C | 134.5±36.7 | 138.1±38.0 | mg/dL |
|  |  |  |  | 214 | 1,089 | Female | HDL-C | 63.7±18.6 | 69.5±18.9 | mg/dL |

GSD: gallstone disease, TC: total cholesterol, TG: triglyceride, LDL-C: low-density lipoprotein cholesterol, HDL-C: high-density lipoprotein cholesterol.
