# Supplementary material for: Blood lipid metabolism and the risk of gallstone disease: a multi-center study and meta-analysis
Source: Lipids Health Dis. 2022 Mar 2;21:26. doi: 10.1186/s12944-022-01635-9 (PMC8889751; doi:10.1186/s12944-022-01635-9)
Supplement: Supplementary file 6 — Additional file 6. The characteristics of the included publications regarding OR and 95%CI of the blood lipid levels on GSD. [file 12944_2022_1635_MOESM6_ESM.docx]

**Additional file 6.** The characteristics of the included publications regarding OR and 95%CI of the blood lipid levels on GSD

| **Study** | **Period** | **Study_design** | **Geographic background** | **Cases** | **Controls** | **Sex** | **Traits** | **Comparison** | **OR (95%CI)** |
| --- | --- | --- | --- | --- | --- | --- | --- | --- | --- |
| Kim, 2021[75] | 2009-2013 | cohort study | Asia | 2,929 | 204,921 | Both | HDL-C | High vs Low | 0.72 (0.54, 0.95) |
|  |  |  |  |  |  | Both | Triglyceride | High vs Low | 0.93 (0.72, 1.21) |
| Sheng, 2020[76] | Aug. 2012 - Jul.2018 | case-control study | Asia | 835 | 835 | Both | Triglyceride | High vs Low | 0.82 (0.71, 0.94) |
|  |  |  |  |  |  | Both | Total cholesterol | High vs Low | 1.29 (1.15, 1.44) |
|  |  |  |  |  |  | Both | LDL-C | High vs Low | 2.63 (2.33, 2.96) |
|  |  |  |  |  |  | Both | HDL-C | High vs Low | 0.14 (0.10, 0.19) |
| Wang, 2020[2] | Jan. 2014 - Jan. 2015 | cross-sectional study | Asia | 168,092 | 1,900,432 | Both | Total cholesterol | High vs Low | 1.04 (1.02, 1.06) |
|  |  |  |  |  |  | Both | Triglyceride | High vs Low | 1.00 (0.99, 1.02) |
|  |  |  |  |  |  | Both | LDL-C | High vs Low | 1.12 (1.09, 1.15) |
|  |  |  |  |  |  | Both | HDL-C | High vs Low | 0.81 (0.79, 0.83) |
| Song, 2020[3] | NA | cross-sectional study | Asia | 274 | 3,735 | Both | Total cholesterol | High vs Low | 1.26 (0.81, 1.95) |
|  |  |  |  |  |  | Both | Triglyceride | High vs Low | 1.35 (0.87, 2.12) |
|  |  |  |  |  |  | Both | LDL-C | High vs Low | 1.21 (0.94, 1.56) |
|  |  |  |  |  |  | Both | HDL-C | High vs Low | 0.89 (0.49, 1.61) |
| Gu, 2020[4] | Jul. 2010 - Dec. 2012 | case-control study | Asia | 94 | 2,194 | Both | Total cholesterol | Per unit | 0.95 (0.83, 1.09) |
|  |  |  |  |  |  | Both | Triglyceride | Per unit | 0.68 (0.41, 1.14) |
|  |  |  |  |  |  | Both | LDL-C | Per unit | 1.92 (1.31, 2.81) |
|  |  |  |  |  |  | Both | HDL-C | Per unit | 1.01 (0.83, 1.23) |
| Kim, 2019[5] | Jan. 2009 - Dec. 2017 | cross-sectional study | Asia | 355 | 7,531 | Both | Total cholesterol | High vs Low | 0.84 (0.59, 1.18) |
|  |  |  |  |  |  | Both | Triglyceride | High vs Low | 1.05 (0.75, 1.47) |
|  |  |  |  |  |  | Both | LDL-C | High vs Low | 1.32 (0.73, 2.41) |
|  |  |  |  |  |  | Both | HDL-C | High vs Low | 0.60 (0.43, 0.83) |
| Kim, 2019[7] | Jun. 2014 - May. 2015 | cross-sectional study | Asia | 806 | 36,495 | Male | Total cholesterol | Per unit | 0.85 (0.70, 1.02) |
|  |  |  |  | 554 | 21,544 | Female | Total cholesterol | Per unit | 0.88 (0.69, 1.12) |
|  |  |  |  | 806 | 36,495 | Male | LDL-C | Per unit | 1.09 (0.90, 1.33) |
|  |  |  |  | 554 | 21,544 | Female | LDL-C | Per unit | 1.07 (0.84, 1.36) |
|  |  |  |  | 806 | 36,495 | Male | HDL-C | Per unit | 0.86 (0.74, 0.99) |
|  |  |  |  | 554 | 21,544 | Female | HDL-C | Per unit | 0.75 (0.57, 0.97) |
| Chang, 2019[77] | 2007 - Dec. 2014 | cohort study | Asia | 104 | 4,735 | Both | Total cholesterol | High vs Low | 1.69 (1.12, 2.55) |
| Dhamnetiya, 2018[8] | Jan. 2013 - Dec. 2013 | case-control study | Asia | 120 | 120 | Both | Total cholesterol | Per unit | 1.01 (1.00, 1.02) |
|  |  |  |  |  |  | Both | Triglyceride | Per unit | 1.01 (1.00, 1.02) |
|  |  |  |  |  |  | Both | LDL-C | Per unit | 1.02 (1.00, 1.03) |
|  |  |  |  |  |  | Both | HDL-C | Per unit | 0.97 (0.93, 1.00) |
| Hu, 2018[78] | Sept. 2012 - Aug. 2013 | cross-sectional study | Asia | 404 | 8,085 | Both | Total cholesterol | High vs Low | 1.05 (0.76, 1.44) |
|  |  |  |  |  |  | Both | Triglyceride | High vs Low | 0.99 (0.76, 1.30) |
|  |  |  |  |  |  | Both | LDL-C | High vs Low | 1.01 (0.73, 1.38) |
|  |  |  |  |  |  | Both | HDL-C | High vs Low | 0.97 (0.73, 1.31) |
| Kwon, 2018[9] | Jan. 2003 - Dec. 2015 | cross-sectional study | Asia | 821 | 19,942 | Both | Total cholesterol | High vs Low | 0.90 (0.72, 1.11) |
|  |  |  |  |  |  | Both | Triglyceride | High vs Low | 1.04 (0.74, 1.45) |
|  |  |  |  |  |  | Both | LDL-C | High vs Low | 1.02 (0.75, 1.38) |
|  |  |  |  |  |  | Both | HDL-C | High vs Low | 0.60 (0.49, 0.74) |
| Shabanzadeh, 2017[79] | Oct. 1982-1992 | cohort study | Europe | 132 | 1,007 | Women | HDL-C | Per unit | 0.99 (0.96, 1.02) |
|  |  |  |  | 132 | 1,007 | Women | Triglyceride | Per unit | 1.06 (0.95, 1.17) |
|  |  |  |  | 103 | 1,124 | Men | HDL-C | Per unit | 0.98 (0.93, 1.03) |
|  |  |  |  | 103 | 1,124 | Men | Triglyceride | Per unit | 1.00 (0.98, 1.02) |
| Kim, 2017[80] | Jan. 2014 - Dec. 2014 | cross-sectional study | Asia | 773 | 17,193 | Male | Triglyceride | High vs Low | 0.96 (0.82, 1.13) |
|  |  |  | Asia | 503 | 12,075 | Female | Triglyceride | High vs Low | 1.55 (1.23, 1.96) |
|  |  |  | Asia | 773 | 17,193 | Male | LDL-C | High vs Low | 0.91 (0.78, 1.07) |
|  |  |  | Asia | 503 | 12,075 | Female | HDL-C | High vs Low | 0.95 (0.76, 1.19) |
| Shabanzadeh, 2016[81] | 1982 - 1993 | cohort study | Europe | 256 | 2,592 | Both | Triglyceride | Per unit | 1.08 (0.97, 1.21) |
|  |  |  |  |  |  | Both | HDL-C | Per unit | 0.82 (0.57, 1.19) |
| Ansari-Moghaddam, 2015[82] | 2012 | cross-sectional study | Asia | 40 | 1,522 | Both | Total cholesterol | High vs Low | 1.14 (0.60, 2.18) |
|  |  |  |  |  |  | Both | Triglyceride | High vs Low | 1.60 (0.79, 3.22) |
|  |  |  |  |  |  | Both | LDL-C | High vs Low | 0.89 (0.46, 1.72) |
|  |  |  |  |  |  | Both | HDL-C | High vs Low | 0.51 (0.26, 0.99) |
| Zhang, 2015[13] | Jan. 2010 - Jan. 2014 | cross-sectional study | Asia | 882 | 9,134 | Both | Total cholesterol | Per unit | 1.02 (1.01, 1.03) |
|  |  |  |  |  |  | Both | Triglyceride | Per unit | 0.89 (0.60, 0.98) |
|  |  |  |  |  |  | Both | LDL-C | Per unit | 1.00 (0.90, 1.02) |
| Dwivedi, 2015[15] | NA | case-control study | Asia | 102 | 256 | Both | Triglyceride | Per unit | 1.01 (1.00, 1.02) |
|  |  |  |  |  |  | Both | HDL-C | Per unit | 0.93 (0.90, 0.95) |
| Chen, 2014[83] | Oct. 2007 - Nov. 2010 | cross-sectional study | Asia | 141 | 1,580 | Both | Total cholesterol | Per unit | 1.00 (0.99, 1.00) |
|  |  |  |  |  |  | Both | Triglyceride | Per unit | 1.00 (0.99, 1.00) |
|  |  |  |  |  |  | Both | LDL-C | Per unit | 1.00 (0.99, 1.01) |
|  |  |  |  |  |  | Both | HDL-C | Per unit | 1.00 (0.98, 1.01) |
| Lin, 2014 | 2011 - 2012 | cross-sectional study | Asia | 734 | 11,180 | Both | Triglyceride | High vs Low | 1.25 (1.05, 1.49) |
|  |  |  |  |  |  | Both | HDL-C | High vs Low | 0.63 (0.54, 0.74) |
|  |  |  |  |  |  | Both | Triglyceride | Per unit | 1.00 (1.00, 1.10) |
|  |  |  |  |  |  | Both | HDL-C | Per unit | 0.99 (0.98, 0.99) |
| Lee, 2014[22] | Jan. 2000 - Aug. 2009 | cross-sectional study | Asia | 768 | 11,265 | Both | Total cholesterol | Per unit | 1.02 (1.00, 1.04) |
|  |  |  |  |  |  | Both | Triglyceride | Per unit | 0.99 (0.98, 1.00) |
|  |  |  |  |  |  | Both | HDL-C | Per unit | 0.92 (0.85, 0.99) |
| Takahashi, 2014[26] | 2010 | cross-sectional study | Asia | 694 | 14,857 | Both | Triglyceride | Per unit | 1.01 (1.01, 1.01) |
|  |  |  |  |  |  | Both | LDL-C | Per unit | 1.00 (1.00, 1.00) |
|  |  |  |  |  |  | Both | HDL-C | Per unit | 1.00 (0.99, 1.00) |
| Xu, 2012[84] | Jan. 2007 - Jun. 2010 | cross-sectional study | Asia | 2,527 | 53,569 | Both | Total cholesterol | High vs Low | 1.04 (0.83, 1.31) |
|  |  |  |  |  |  | Both | Triglyceride | High vs Low | 0.93 (0.82, 1.06) |
|  |  |  |  |  |  | Both | LDL-C | High vs Low | 0.87 (0.74, 1.03) |
|  |  |  |  |  |  | Both | HDL-C | High vs Low | 0.93 (0.77, 1.14) |
| Kim, 2011[28] | Jan. 2006 - Dec. 2007 | cross-sectional study | Asia | 173 | 3,952 | Female | HDL-C | High vs Low | 0.55 (0.33, 0.93) |
| Krawczyk, 2011[85] | NA | case-control study | Europe | 229 | 258 | Both | Total cholesterol | Per unit | 0.99 (0.98, 1.00) |
|  |  |  |  |  |  | Both | Triglyceride | Per unit | 1.00 (1.00, 1.01) |
|  |  |  |  |  |  | Both | LDL-C | Per unit | 0.99 (0.99, 1.00) |
|  |  |  |  |  |  | Both | HDL-C | Per unit | 0.99 (0.97, 1.00) |
| Banim, 2011[86] | 1993 and 1997 - Jun. 2007 | cohort study | America | 95 | 11,093 | Male | Total cholesterol | High vs Low | 0.68 (0.36, 1.25) |
|  |  |  |  | 201 | 12,874 | Female | Total cholesterol | High vs Low | 1.14 (0.70, 1.72) |
|  |  |  |  | 95 | 11,093 | Male | Triglyceride | High vs Low | 2.02 (1.03, 3.98) |
|  |  |  |  | 201 | 12,874 | Female | Triglyceride | High vs Low | 2.43 (1.52, 3.90) |
|  |  |  |  | 201 | 12,874 | Female | LDL-C | High vs Low | 1.13 (0.72, 1.77) |
|  |  |  |  | 95 | 11,093 | Male | LDL-C | High vs Low | 1.05 (0.56, 1.95) |
|  |  |  |  | 201 | 12,874 | Female | HDL-C | High vs Low | 0.55 (0.36, 0.85) |
|  |  |  |  | 95 | 11,093 | Male | HDL-C | High vs Low | 0.22 (0.09, 0.52) |
| Wang, 2010[30] | Jan. 2008 - Jul. 2008 | case-control study | Asia | 100 | 147 | Both | Total cholesterol | Per unit | 1.02 (1.01, 1.04) |
|  |  |  |  |  |  | Both | Triglyceride | Per unit | 1.01 (0.99, 1.02) |
| Halldestam, 2009[87] | NA | cohort study | Europe | 42 | 503 | Both | Triglyceride | Per unit | 0.90 (0.42, 1.90) |
|  |  |  |  |  |  | Both | LDL-C | Per unit | 1.59 (1.32, 1.91) |
|  |  |  |  |  |  | Both | HDL-C | Per unit | 1.29 (0.49, 3.42) |
| Walcher, 2009[88] | Nov. 2002 - Dec. 2002 | cross-sectional study | Europe | 167 | 1,962 | Both | Total cholesterol | Per unit | 0.65 (0.52, 0.79) |
|  |  |  |  |  |  | Both | Triglyceride | Per unit | 0.91 (0.79, 1.04) |
|  |  |  |  |  |  | Both | LDL-C | Per unit | 0.69 (0.55, 0.86) |
|  |  |  |  |  |  | Both | HDL-C | Per unit | 0.63 (0.39, 1.03) |
| Sun, 2009[89] | Jan. 2007 - Dec. 2007 | cross-sectional study | Asia | 384 | 3,189 | Both | Total cholesterol | High vs Low | 1.70 (1.20, 2.43) |
|  |  |  |  |  |  | Both | Triglyceride | High vs Low | 1.67 (1.31, 2.13) |
|  |  |  |  |  |  | Both | LDL-C | High vs Low | 1.71 (1.09, 2.68) |
|  |  |  |  |  |  | Both | HDL-C | High vs Low | 0.48 (0.31, 0.74) |
| Tirziu, 2008[32] | Nov. 2002 - Sept.2007 | case-control study | Europe | 109 | 271 | Both | HDL-C | Per unit | 0.98 (0.96, 1.00) |
| Festi, 2008[90] | 1985 and 1988 - 10 years | cross-sectional study | Europe | 485 | 9,032 | Both | Total cholesterol | Per unit | 1.00 (0.99, 1.00) |
|  |  |  |  |  |  | Both | Triglyceride | Per unit | 1.00 (1.00, 1.00) |
|  |  |  |  |  |  | Both | HDL-C | Per unit | 0.99 (0.98, 1.00) |
| Andreotti, 2008[35] | Jun. 1997 - May. 2001 | case-control study | Asia | 981 | 858 | Both | Total cholesterol | High vs Low | 0.84 (0.54, 1.31) |
|  |  |  |  |  |  | Both | Triglyceride | High vs Low | 1.43 (1.08, 1.90) |
|  |  |  |  |  |  | Both | LDL-C | High vs Low | 0.91 (0.59, 1.42) |
|  |  |  |  |  |  | Both | HDL-C | High vs Low | 0.44 (0.30, 0.64) |
| Chen, 2006[91] | Aug. 2003 - Apr. 2004 | cross-sectional study | Asia | 74 | 1,518 | Male | Total cholesterol | High vs Low | 0.52 (0.16, 1.52) |
|  |  |  |  | 94 | 1,647 | Female | Total cholesterol | High vs Low | 2.19 (1.18, 4.05) |
|  |  |  |  | 94 | 1,647 | Female | Triglyceride | High vs Low | 2.12 (1.29, 3.46) |
|  |  |  |  | 74 | 1,518 | Male | Triglyceride | High vs Low | 1.16 (0.66, 2.03) |
| Liu, 2006[40] | Jan. 2002 - Dec. 2007 | cross-sectional study | Asia | 126 | 2,260 | Both | Total cholesterol | High vs Low | 0.99 (0.64, 1.53) |
|  |  |  |  |  |  | Both | Triglyceride | High vs Low | 1.25 (0.75, 2.08) |
|  |  |  |  |  |  | Both | HDL-C | High vs Low | 0.50 (0.17, 1.44) |
| Nervi, 2006[41] | 1993 - 2000 | case-control study | America | 299 | 582 | Female | Triglyceride | High vs Low | 0.83 (0.63, 1.25) |
|  |  |  |  |  |  | Female | HDL-C | High vs Low | 0.67 (0.50, 0.91) |
| Sakuta, 2005[43] | NA | cross-sectional study | Asia | 39 | 926 | Male | Total cholesterol | Per unit | 0.95 (0.69, 1.31) |
|  |  |  |  |  |  | Male | Triglyceride | Per unit | 0.99 (0.72, 1.36) |
| Mendez-Sanchez, 2005[45] | NA | cross-sectional study | America | 65 | 180 | Both | HDL-C | High vs Low | 0.43 (0.20, 0.95) |
|  |  |  |  |  |  | Both | Triglyceride | High vs Low | 1.46 (0.78, 2.74) |
|  |  |  |  |  |  | Both | Total cholesterol | High vs Low | 1.20 (0.59, 2.47) |
|  |  |  |  |  |  | Both | LDL-C | High vs Low | 1.22 (0.63, 2.38) |
| Volzke, 2005[46] | NA | cross-sectional study | Europe | 305 | 1,747 | Male | LDL-C | Per unit | 0.88 (0.77, 0.99) |
|  |  |  |  | 586 | 1,564 | Female | LDL-C | Per unit | 0.85 (0.76, 0.94) |
|  |  |  |  | 305 | 1,747 | Male | HDL-C | Per unit | 0.66 (0.44, 1.00) |
|  |  |  |  | 586 | 1,564 | Female | HDL-C | Per unit | 0.76 (0.58, 0.99) |
| Boland, 2002[92] | 1987 - 1996 | cohort study | America | 179 | 5,660 | Male | Total cholesterol | High vs Low | 0.91 (0.60, 1.40) |
|  |  |  |  | 370 | 6,564 | Female | Total cholesterol | High vs Low | 1.07 (0.80, 1.50) |
|  |  |  |  | 179 | 5,660 | Male | Triglyceride | High vs Low | 1.65 (1.00, 2.70) |
|  |  |  |  | 370 | 6,564 | Female | Triglyceride | High vs Low | 2.57 (1.70, 3.90) |
|  |  |  |  | 370 | 6,564 | Female | LDL-C | High vs Low | 0.99 (0.70, 1.40) |
|  |  |  |  | 179 | 5,660 | Male | LDL-C | High vs Low | 0.85 (0.50, 1.40) |
|  |  |  |  | 179 | 5,660 | Male | HDL-C | High vs Low | 0.42 (0.30, 0.70) |
|  |  |  |  | 370 | 6,564 | Female | HDL-C | High vs Low | 0.64 (0.50, 0.90) |
| Brasca, 2000[93] | NA | cross-sectional study | America | 169 | 539 | Female | Total cholesterol | High vs Low | 0.80 (0.50, 1.20) |
|  |  |  |  | 72 | 393 | Male | Total cholesterol | High vs Low | 1.00 (0.50, 1.90) |
|  |  |  |  | 72 | 393 | Male | Triglyceride | High vs Low | 0.80 (0.40, 1.80) |
|  |  |  |  | 169 | 539 | Female | Triglyceride | High vs Low | 1.90 (1.00, 3.60) |
|  |  |  |  | 72 | 393 | Male | LDL-C | High vs Low | 0.90 (0.50, 1.70) |
|  |  |  |  | 169 | 539 | Female | LDL-C | High vs Low | 0.80 (0.50, 1.20) |
|  |  |  |  | 72 | 393 | Male | HDL-C | High vs Low | 0.60 (0.20, 1.70) |
|  |  |  |  | 169 | 539 | Female | HDL-C | High vs Low | 0.90 (0.50, 1.50) |
| Misciagna, 2000[53] | May. 1985 - Jun. 1993 | case-control study | Europe | 84 | 271 | Both | Total cholesterol | High vs Low | 0.83 (0.37, 1.88) |
|  |  |  |  |  |  | Both | Triglyceride | High vs Low | 2.94 (1.17, 7.36) |
|  |  |  |  |  |  | Both | HDL-C | High vs Low | 0.66 (0.29, 1.47) |
|  |  |  |  |  |  | Both | Total cholesterol | Per unit | 0.60 (0.39, 0.93) |
|  |  |  |  |  |  | Both | Triglyceride | Per unit | 1.72 (0.97, 3.05) |
| Attili, 1997[94] | Dec. 1984 - Apr. 1987 | cohort study | Europe | 1,511 | 14,399 | Male | Total cholesterol | High vs Low | 0.66 (0.57, 0.78) |
|  |  |  |  | 2,584 | 11,090 | Female | Total cholesterol | High vs Low | 0.77 (0.68, 0.88) |
|  |  |  |  | 1,511 | 14,399 | Male | Triglyceride | High vs Low | 0.94 (0.80, 1.09) |
|  |  |  |  | 2,584 | 11,090 | Female | Triglyceride | High vs Low | 1.31 (1.15, 1.49) |
|  |  |  |  | 2,584 | 11,090 | Female | HDL-C | High vs Low | 0.70 (0.62, 0.79) |
|  |  |  |  | 1,511 | 14,399 | Male | HDL-C | High vs Low | 0.73 (0.63, 0.85) |
| Villalpando, 1997[66] | NA | cross-sectional study | America | 19 | 920 | Male | Total cholesterol | High vs Low | 0.41 (0.05, 1.76) |
|  |  |  |  | 124 | 1,211 | Female | Total cholesterol | High vs Low | 1.27 (0.81, 1.99) |
|  |  |  |  | 19 | 920 | Male | Triglyceride | High vs Low | 1.01 (0.35, 3.28) |
|  |  |  |  | 124 | 1,211 | Female | Triglyceride | High vs Low | 1.57 (1.05, 2.36) |
| Shinchi, 1993[67] | Oct. 1986 - Dec. 1990 | case-control study | Asia | 61 | 2,494 | Male | Total cholesterol | High vs Low | 0.40 (0.20, 0.90) |
|  |  |  |  |  |  | Male | Triglyceride | High vs Low | 1.20 (0.50, 2.60) |
|  |  |  |  |  |  | Male | LDL-C | High vs Low | 0.50 (0.30, 1.20) |
|  |  |  |  |  |  | Male | HDL-C | High vs Low | 1.00 (0.40, 2.30) |
| Loria, 1994[68] | Nov. 1985 - Apr. 1986 | cross-sectional study | Europe | 61 | 1,804 | Both | Total cholesterol | High vs Low | 1.14 (0.78, 1.66) |
|  |  |  |  |  |  | Both | Triglyceride | High vs Low | 1.03 (0.65, 1.63) |
|  |  |  |  |  |  | Both | HDL-C | High vs Low | 0.91 (0.60, 1.39) |
| Petitti, 1981[95] | NA | cross-sectional study | America | 65 | 803 | Female | LDL-C | Per unit | 1.00 (0.90, 1.10) |
|  |  |  |  |  |  | Female | HDL-C | Per unit | 0.80 (0.60, 1.00) |
| Scragg, 1984[71] | Dec. 1978 - Sept. 1980 | case-control study | Oceania | 46 | 102 | Male | Total cholesterol | Per unit | 0.64 (0.41, 0.99) |
|  |  |  |  | 127 | 182 | Female | Total cholesterol | Per unit | 0.79 (0.61, 1.03) |
|  |  |  |  | 124 | 175 | Female | Triglyceride | Per unit | 2.94 (1.38, 6.25) |
|  |  |  |  | 44 | 99 | Male | Triglyceride | Per unit | 1.48 (1.00, 2.18) |
| GREPCO, 1988[72] | NA | cross-sectional study | Europe | 65 | 1,137 | Male | Triglyceride | Per unit | 1.01 (1.00, 1.03) |
|  |  |  |  | 66 | 979 | Female | Triglyceride | Per unit | 1.01 (1.00, 1.01) |
| Nomura, 1988[96] | May. 1984 - Dec. 1984 | cross-sectional study | Asia | 82 | 1,760 | Both | Total cholesterol | High vs Low | 1.60 (0.80, 3.20) |
|  |  |  |  |  |  | Both | Triglyceride | High vs Low | 0.60 (0.30, 1.20) |
| Jorgensen, 1989[97] | Nov. 1982 - Feb. 1984 | cross-sectional study | Europe | 280 | 3,128 | Both | Total cholesterol | Per unit | 0.87 (0.77, 0.99) |
|  |  |  |  |  |  | Both | Triglyceride | Per unit | 1.09 (0.97, 1.22) |
|  |  |  |  |  |  | Both | LDL-C | Per unit | 0.88 (0.77, 1.00) |
|  |  |  |  |  |  | Both | HDL-C | Per unit | 0.72 (0.50, 1.04) |
| Thijs, 1990[98] | 1983 - 1985 | case-control study | Europe | 250 | 526 | Both | Total cholesterol | Per unit | 0.71 (0.61, 0.83) |
|  |  |  |  |  |  | Both | Triglyceride | Per unit | 3.77 (2.21, 6.44) |
|  |  |  |  |  |  | Both | HDL-C | Per unit | 0.14 (0.07, 0.28) |
| Sichieri, 1990[99] | 1971 - 1984 | cohort study | America | 368 | 10,551 | Both | Total cholesterol | High vs Low | 1.12 (1.02, 1.22) |
| Maurer, 1990[100] | 1982 - 1984 | cross-sectional study | America | 253 | 1,072 | Female | Total cholesterol | Per unit | 0.80 (0.70, 0.90) |
|  |  |  |  | 53 | 915 | Male | Total cholesterol | Per unit | 1.00 (0.70, 1.30) |
|  |  |  |  | 53 | 915 | Male | HDL-C | Per unit | 0.80 (0.60, 1.00) |
|  |  |  |  | 253 | 1,072 | Female | HDL-C | Per unit | 1.00 (0.90, 1.20) |
| Mohr, 1991[74] | 1984 - 1987 | cross-sectional study | America | 216 | 1,087 | Female | Triglyceride | Per unit | 5.29 (4.45, 6.12) |
|  |  |  |  |  |  | Female | LDL-C | Per unit | 0.99 (0.99, 1.00) |
|  |  |  |  |  |  | Female | HDL-C | Per unit | 1.00 (0.98, 1.01) |
| Kato, 1992[101] | 1900 - 1919 | cohort study | America | 471 | 7,381 | Both | Total cholesterol | High vs Low | 0.90 (0.70, 1.20) |
|  |  |  |  |  |  | Both | Triglyceride | High vs Low | 1.40 (1.10, 1.90) |

LDL-C: low-density lipoprotein cholesterol, HDL-C: high-density lipoprotein cholesterol
