# Supplementary material for: Blood lipid metabolism and the risk of gallstone disease: a multi-center study and meta-analysis
Source: Lipids Health Dis. 2022 Mar 2;21:26. doi: 10.1186/s12944-022-01635-9 (PMC8889751; doi:10.1186/s12944-022-01635-9)
Supplement: Supplementary file 7 — Additional file 7. Quality assessment of the included studies. [file 12944_2022_1635_MOESM7_ESM.docx]

**Additional file 7.** Quality assessment of the included studies.

| **Study** | **Study_design** | **Score^a^** | **Grade** |  | **Study** | **Study_design** | **Score^a^** | **Grade** |  | **Study** | **Study_design** | **Score^a^** | **Grade** |
| --- | --- | --- | --- | --- | --- | --- | --- | --- | --- | --- | --- | --- | --- |
| Kim, 2021 | cohort study | 8 | High |  | Takahashi, 2014 | cross-sectional study | 9 | High |  | Boland, 2002 | cohort study | 7 | High |
| Sheng, 2020 | case-control study | 7 | High |  | Chen, 2012 | cross-sectional study | 7 | Moderate |  | Devesa, 2001 | cross-sectional study | 7 | Moderate |
| Sepehrimanesh, 2020 | cross-sectional study | 8 | High |  | Xu, 2012 | cross-sectional study | 9 | High |  | Brasca, 2000 | cross-sectional study | 6 | Moderate |
| Wang, 2020 | cross-sectional study | 7 | Moderate |  | Kim, 2011 | cross-sectional study | 8 | High |  | Misciagna, 2000 | case-control study | 4 | Moderate |
| Song, 2020 | cross-sectional study | 10 | High |  | Krawczyk, 2011 | case-control study | 6 | Moderate |  | Han, 2000 | case-control study | 5 | Moderate |
| Gu, 2020 | cross-sectional study | 7 | Moderate |  | Banim, 2011 | cohort study | 8 | High |  | Chen, 1999 | case-control study | 5 | Moderate |
| Kim, 2019 | cross-sectional study | 8 | High |  | Karayalcin, 2010 | cross-sectional study | 8 | High |  | Duque, 1999 | cross-sectional study | 8 | High |
| Hayat, 2019 | cross-sectional study | 7 | Moderate |  | Wang, 2010 | case-control study | 6 | Moderate |  | Sasazuki, 1999 | cross-sectional study | 8 | High |
| Kim, 2019 | cross-sectional study | 8 | High |  | Siddapuram, 2010 | case-control study | 4 | Moderate |  | Niemi, 1999 | cross-sectional study | 6 | Moderate |
| Chang, 2019 | cohort study | 8 | High |  | Halldestam, 2009 | cohort study | 7 | High |  | Chen, 1998 | cross-sectional study | 7 | Moderate |
| Dhamnetiya, 2018 | case-control study | 6 | Moderate |  | Walcher, 2009 | cross-sectional study | 9 | High |  | Borch, 1998 | cross-sectional study | 7 | Moderate |
| Hu, 2018 | cross-sectional study | 9 | High |  | Sun, 2009 | cross-sectional study | 8 | High |  | Fu, 1997 | case-control study | 4 | Moderate |
| Kwon, 2018 | cross-sectional study | 9 | High |  | Tirziu, 2008 | case-control study | 5 | Moderate |  | Miquel, 1998 | cross-sectional study | 5 | Moderate |
| Shabanzadeh, 2017 | cohort study | 8 | High |  | Festi, 2008 | cross-sectional study | 10 | High |  | Singh, 1997 | cross-sectional study | 5 | Moderate |
| Serin, 2017 | cross-sectional study | 9 | High |  | Chang, 2008 | cross-sectional study | 8 | High |  | Attili, 1997 | cross-sectional study | 8 | High |
| Kim, 2017 | cross-sectional study | 8 | High |  | Kuo, 2008 | cross-sectional study | 7 | Moderate |  | Tang, 1996 | cross-sectional study | 6 | Moderate |
| Ravikanth, 2016 | case-control study | 5 | Moderate |  | Andreotti, 2008 | case-control study | 6 | Moderate |  | Bertomeu, 1996 | case-control study | 4 | Moderate |
| Shabanzadeh, 2016 | cohort study | 8 | High |  | Wang, 2007 | case-control study | 6 | Moderate |  | Villalpando, 1997 | cross-sectional study | 5 | Moderate |
| Zhan, 2016 | cross-sectional study | 5 | Moderate |  | Mella, 2007 | case-control study | 5 | Moderate |  | Shinchi, 1993 | case-control study | 6 | Moderate |
| Ansari, 2015 | cross-sectional study | 8 | High |  | Acalovschi, 2006 | case-control study | 4 | Moderate |  | Loria, 1994 | cross-sectional study | 8 | High |
| Zhang, 2015 | cross-sectional study | 8 | High |  | Chen, 2006 | cross-sectional study | 8 | High |  | Juvonen, 1995 | case-control study | 5 | Moderate |
| Dai, 2015 | case-control study | 4 | Moderate |  | Méndez-Sánchez, 2006 | cross-sectional study | 8 | High |  | Sarin, 1995 | cross-sectional study | 6 | Moderate |
| Dwivedi, 2015 | case-control study | 5 | Moderate |  | Liu, 2006 | cross-sectional study | 6 | Moderate |  | Petitti, 1981 | cross-sectional study | 5 | Moderate |
| Kwak, 2015 | cross-sectional study | 8 | High |  | Nervi, 2006 | case-control study | 6 | Moderate |  | Scragg, 1984 | case-control study | 4 | Moderate |
| Martinez-Lopez, 2015 | case-control study | 5 | Moderate |  | Wang, 2006 | case-control study | 5 | Moderate |  | GREPCO, 1988 | cross-sectional study | 5 | Moderate |
| Sarac, 2015 | case-control study | 6 | Moderate |  | Sakuta, 2005 | cross-sectional study | 5 | Moderate |  | Mellstrom, 1988 | cross-sectional study | 4 | Moderate |
| Chen, 2014 | cross-sectional study | 9 | High |  | Méndez-Sánchez, 2005 | case-control study | 4 | Moderate |  | Nomura, 1988 | cross-sectional study | 6 | Moderate |
| Zamani, 2014 | cross-sectional study | 8 | High |  | Mendez-Sanchez, 2005 | cross-sectional study | 6 | Moderate |  | Jorgensen, 1989 | cross-sectional study | 7 | Moderate |
| Zhu, 2014 | cross-sectional study | 7 | Moderate |  | Volzke, 2005 | cross-sectional study | 9 | High |  | Thijs, 1990 | case-control study | 5 | Moderate |
| Lin, 2014 | cross-sectional study | 8 | High |  | Jiang, 2004 | case-control study | 5 | Moderate |  | Sichieri, 1990 | cohort study | 6 | Moderate |
| Lee, 2014 | cross-sectional study | 9 | High |  | Galman, 2004 | cross-sectional study | 9 | High |  | Maurer, 1990 | cross-sectional study | 8 | High |
| Ajdarkosh, 2013 | case-control study | 4 | Moderate |  | Hasegawa, 2003 | case-control study | 4 | Moderate |  | Mohr, 1991 | cross-sectional study | 9 | High |
| Chen, 2014 | cohort study | 7 | High |  | Gustafsson, 2003 | case-control study | 5 | Moderate |  | Kato, 1992 | cohort study | 6 | Moderate |
| Batajoo, 2013 | cross-sectional study | 7 | Moderate |  | Kurtul, 2002 | case-control study | 4 | Moderate |  |  |  |  |  |

**^a^** Quality assessment were conducted for the included cohort and case-control studies by the Newcastle-Ottawa scale, and the Agency for Healthcare Research Quality (AHRQ) for cross-sectional studies.
