# Supplementary material for: Blood lipid metabolism and the risk of gallstone disease: a multi-center study and meta-analysis
Source: Lipids Health Dis. 2022 Mar 2;21:26. doi: 10.1186/s12944-022-01635-9 (PMC8889751; doi:10.1186/s12944-022-01635-9)
Supplement: Supplementary file 8 — Additional file 8. Subgroup analysis and meta-regression in meta-analysis. [file 12944_2022_1635_MOESM8_ESM.docx]

**Additional file 8.** Subgroup analysis and meta-regression in meta-analysis.

|  | **Standardized Mean Difference** | | | | | **Odd Ratio (High vs Low)** | | | | | **Odd Ratio (Per Unit)** | | | | |
| --- | --- | --- | --- | --- | --- | --- | --- | --- | --- | --- | --- | --- | --- | --- | --- |
|  | **No.^a^** | **SMD (95%CI)** | ***P*** | ***I^2^*** | ***P-for Metareg^b^*** | **No.^a^** | **OR (95%CI)** | ***P*** | ***I^2^*** | ***P-for Metareg^b^*** | **No.^a^** | **OR (95%CI)** | ***P*** | ***I^2^*** | ***P-for Metareg^b^*** |
| **Total cholesterol** | **85** | **0.040 (0.009, 0.071)** | **0.012** | **90.9%** |  | **34** | **0.974 (0.896, 1.059)** | **0.539** | **76.5%** |  | **22** | **0.991 (0.980, 1.002)** | **0.125** | **84.6%** |  |
| **Study_design** |  |  |  |  | 0.832 |  |  |  |  | 0.442 |  |  |  |  | 0.725 |
| cross-sectional study | 49 | 0.081 (0.051, 0.111) | 1.44×10^-07^ | 88.2% |  | 21 | 0.993 (0.891, 1.107) | 0.901 | 63.9% |  | 15 | 0.991 (0.964, 1.018) | 0.049 | 82.7% |  |
| case-control study | 33 | 0.009 (-0.129, 0.147) | 0.899 | 91.7% |  | 4 | 0.846 (0.523, 1.369) | 0.497 | 76.9% |  | 7 | 0.985 (0.971, 1.000) | 0.506 | 89.1% |  |
| cohort study | 2 | 0.148 (0.075, 0.220) | 7.19×10^-05^ | 0.0% |  | 9 | 0.944 (0.777, 1.147) | 0.562 | 85.1% |  |  |  |  |  |  |
| **Geographic background** | | |  |  | 0.342 |  |  |  |  | 0.142 |  |  |  |  | 0.108 |
| Asia | 45 | 0.059 (0.027, 0.091) | 3.44×10^-04^ | 91.9% |  | 19 | 1.014 (0.905, 1.137) | 0.81 | 72.8% |  | 12 | 0.998 (0.983, 1.013) | 0.771 | 79.0% |  |
| Europe | 22 | -0.05 (-0.194, 0.094) | 0.495 | 84.9% |  | 4 | 0.780 (0.644, 0.945) | 0.011 | 59.9% |  | 6 | 0.979 (0.960, 1.000) | 0.047 | 89.6% |  |
| America | 14 | -0.087 (-0.303, 0.130) | 0.431 | 90.6% |  | 11 | 1.074 (0.996, 1.158) | 0.063 | 0.0% |  | 2 | 0.854 (0.700, 1.041) | 0.118 | 41.7% |  |
| **Gender** |  |  |  |  | 0.396 |  |  |  |  | 0.127 |  |  |  |  | 0.258 |
| Female | 18 | 0.094 (-0.059, 0.247) | 0.228 | 87.2% |  | 6 | 1.060 (0.807, 1.391) | 0.677 | 72.2% |  | 3 | 0.812 (0.733, 0.900) | 6.90×10^-05^ | 0.0% |  |
| Male | 12 | -0.058 (-0.153, 0.038) | 0.236 | 52.6% |  | 7 | 0.680 (0.593, 0.779) | 2.76×10^-08^ | 0.0% |  | 4 | 0.868 (0.756, 0.998) | 0.047 | 2.2% |  |
| **Quality of included studies** | | |  |  | 0.440 |  |  |  |  | 0.960 |  |  |  |  | 0.770 |
| High | 28 | 0.084 (0.050, 0.118) | 1.37×10^-06^ | 81.6% |  | 21 | 0.988 (0.861, 1.135) | 0.867 | 81.8% |  | 12 | 0.988 (0.973, 1.002) | 0.103 | 85.5% |  |
| Moderate | 57 | 0.010 (-0.071, 0.090) | 0.813 | 92.7% |  | 13 | 1.034 (0.958, 1.117) | 0.39 | 25.0% |  | 10 | 0.983 (0.957, 1.010) | 0.224 | 85.1% |  |
|  |  |  |  |  |  |  |  |  |  |  |  |  |  |  |  |
| **Triglycerides** | **82** | **0.302 (0.251, 0.354)** | **1.32×10^-30^** | **97.7%** |  | **37** | **1.192 (1.097, 1.295)** | **3.47×10^-05^** | **75.8%** |  | **28** | **1.011 (1.006, 1.016)** | **5.12×10^-05^** | **96.0%** |  |
| **Study_design** |  |  |  |  | 0.908 |  |  |  |  | 0.052 |  |  |  |  | 0.779 |
| cross-sectional study | 48 | 0.252 (0.189, 0.314) | 2.38×10^-15^ | 98.4% |  | 26 | 1.143 (1.094, 1.254) | 0.002 | 69.3% |  | 16 | 1.012 (1.006, 1.019) | 2.86×10^-04^ | 97.7% |  |
| case-control study | 33 | 0.455 (0.279, 0.631) | 3.98×10^-07^ | 94.9% |  | 5 | 1.126 (0.791, 1.602) | 0.51 | 78.7% |  | 8 | 1.010 (0.996, 1.024) | 0.156 | 81.5% |  |
| cohort study | 1 | 0.043 (-0.369, 0.455) | 0.838 | NA |  | 6 | 1.677 (1.176, 2.390) | 0.004 | 79.5% |  | 4 | 1.004 (0.985, 1.024) | 0.663 | 0.0% |  |
| **Geographic background** | | |  |  | 0.749 |  |  |  |  | 0.874 |  |  |  |  | 0.857 |
| Asia | 46 | 0.126 (0.095, 0.158) | 3.84×10^-15^ | 91.3% |  | 22 | 1.101 (1.008, 1.201) | 0.032 | 69.7% |  | 13 | 1.011 (1.004, 1.018) | 0.002 | 79.2% |  |
| Europe | 21 | 0.398 (0.091, 0.704) | 0.011 | 95.2% |  | 4 | 1.181 (0.889, 1.569) | 0.251 | 79.5% |  | 12 | 1.006 (0.999, 1.012) | 0.088 | 80.2% |  |
| America | 12 | 1.356 (0.308, 2.405) | 0.011 | 99.5% |  | 11 | 1.537 (1.205, 1.960) | 0.001 | 62.1% |  | 1 | 5.290 (4.511, 6.204) | 2.64×10^-93^ | NA |  |
| Oceania |  |  |  |  |  |  |  |  |  |  | 2 | 1.925 (0.998, 3.711) | 0.051 | 60.4% |  |
| **Gender** |  |  |  |  | 0.460 |  |  |  |  | 0.013 |  |  |  |  | 0.350 |
| Female | 16 | 1.295 (0.338, 2.251) | 0.008 | 99.4% |  | 8 | 1.621 (1.285, 2.045) | 4.70×10^-05^ | 74.6% |  | 4 | 1.961 (1.021, 3.764) | 0.043 | 99.3% |  |
| Male | 11 | 0.159 (-0.057, 0.375) | 0.148 | 88.4% |  | 8 | 1.05 (0.898, 1.227) | 0.497 | 27.7% |  | 4 | 1.006 (0.988, 1.026) | 0.501 | 30.8% |  |
| **Quality of included studies** | | |  |  | 0.418 |  |  |  |  | 0.984 |  |  |  |  | 0.679 |
| High | 25 | 0.467 (0.338, 0.597) | 1.32×10^-12^ | 99.1% |  | 24 | 1.210 (1.077, 1.359) | 0.001 | 78.1% |  | 15 | 1.012 (1.005, 1.020) | 0.001 | 97.7% |  |
| Moderate | 57 | 0.296 (0.220, 0.372) | 1.82×10^-14^ | 92.7% |  | 13 | 1.207 (1.011, 1.443) | 0.038 | 62.0% |  | 13 | 1.010 (1.001, 1.018) | 0.026 | 75.1% |  |
|  |  |  |  |  |  |  |  |  |  |  |  |  |  |  |  |
| **LDL-C** | **59** | **0.059 (0.027, 0.092)** | **3.85×10^-04^** | **91.6%** |  | **21** | **1.054 (0.912, 1.219)** | **0.473** | **93.0%** |  | **17** | **0.998 (0.987, 1.008)** | **0.802** | **81.4%** |  |
| **Study_design** |  |  |  |  | 0.629 |  |  |  |  | 0.782 |  |  |  |  | 0.779 |
| cross-sectional study | 37 | 0.079 (0.057, 0.107) | 5.33×10^-08^ | 85.8% |  | 14 | 0.996 (0.898, 1.105) | 0.939 | 80.7% |  | 14 | 0.995 (0.983, 1.007) | 0.416 | 75.4% |  |
| case-control study | 22 | 0.086 (-0.097, 0.269) | 0.357 | 94.4% |  | 3 | 1.106 (0.401, 3.045) | 0.846 | 95.1% |  | 2 | 1.003 (0.979, 1.028) | 0.796 | 87.7% |  |
| cohort study |  |  |  |  |  | 4 | 1.002 (0.799, 1.255) | 0.988 | 0.0% |  | 1 | 1.590 (1.322, 1.913) | 8.65×10^-07^ | NA |  |
| **Geographic background** | | |  |  | 0.254 |  |  |  |  | 0.957 |  |  |  |  | 0.857 |
| Asia | 35 | 0.040 (0.004, 0.076) | 0.029 | 93.7% |  | 14 | 1.084 (0.912, 1.289) | 0.729 | 82.6% |  | 9 | 1.009 (0.995, 1.024) | 0.217 | 67.9% |  |
| Europe | 11 | 0.081 (-0.012, 0.174) | 0.089 | 40.1% |  |  |  |  |  |  | 6 | 0.947 (0.829, 1.081) | 0.417 | 90.0% |  |
| America | 11 | 0.126 (-0.052, 0.306) | 0.165 | 82.3% |  | 7 | 0.968 (0.806, 1.169) | 0.361 | 0.0% |  | 2 | 0.990 (0.985, 0.995) | 8.86×10^-05^ | 0.0% |  |
| **Gender** |  |  |  |  | 0.694 |  |  |  |  | 0.134 |  |  |  |  | 0.350 |
| Female | 11 | 0.141 (0.023, 0.259) | 0.02 | 67.9% |  | 3 | 0.966 (0.765, 1.219) | 0.594 | 0.0% |  | 4 | 0.963 (0.893, 1.039) | 0.332 | 63.8% |  |
| Male | 7 | -0.018 (-0.115, 0.079) | 0.719 | 42.1% |  | 5 | 0.893 (0.776, 1.026) | 0.409 | 0.0% |  | 2 | 0.968 (0.784, 1.197) | 0.766 | 70.6% |  |
| **Quality of included studies** | | |  |  | 0.930 |  |  |  |  | 0.294 |  |  |  |  | 0.679 |
| High | 18 | 0.079 (0.044, 0.113) | 6.67×10^-06^ | 80.7% |  | 15 | 1.102 (0.880, 1.382) | 0.397 | 94.9% |  | 12 | 0.998 (0.984, 1.011) | 0.745 | 82.4% |  |
| Moderate | 41 | 0.074 (-0.014, 0.162) | 0.101 | 93.1% |  | 6 | 0.951 (0.765, 1.182) | 0.652 | 43.3% |  | 5 | 1.001 (0.967, 1.036) | 0.976 | 82.6% |  |
|  |  |  |  |  |  |  |  |  |  |  |  |  |  |  |  |
| **HDL-C** | **76** | **-0.182 (-0.217, -0.147)** | **3.67×10^-24^** | **93.9%** |  | **30** | **0.636 (0.570, 0.710)** | **6.22×10^-15^** | **85.0%** |  | **27** | **0.974 (0.961, 0.987)** | **6.07×10^-05^** | **76.4%** |  |
| **Study_design** |  |  |  |  | 0.056 |  |  |  |  | 0.020 |  |  |  |  | 0.970 |
| cross-sectional study | 46 | -0.119 (-0.147, -0.090) | 2.21×10^-16^ | 87.8% |  | 20 | 0.732 (0.677, 0.791) | 4.36×10^-15^ | 62.0% |  | 19 | 0.983 (0.970, 0.995) | 0.007 | 65.9% |  |
| case-control study | 29 | -0.467 (-0.685, -0.250) | 2.55×10^-05^ | 96.5% |  | 4 | 0.501 (0.270, 0.929) | 0.028 | 93.1% |  | 6 | 0.965 (0.929, 1.001) | 0.059 | 89.2% |  |
| cohort study | 1 | 0.092 (-0.321, 0.504) | 0.663 | NA |  | 6 | 0.487 (0.349, 0.680 | 2.38×10^-05^ | 54.7% |  | 2 | 0.868 (0.615, 1.225) | 0.42 | 0.0% |  |
| **Geographic background** | | |  |  | 0.017 |  |  |  |  | 0.832 |  |  |  |  | 0.818 |
| Asia | 41 | -0.170 (-0.202, -0.137) | 3.67×10^-24^ | 92.6% |  | 18 | 0.635 (0.545, 0.740) | 5.89×10^-09^ | 89.6% |  | 12 | 0.970 (0.955, 0.986) | 3.44×10^-04^ | 79.9% |  |
| Europe | 19 | -0.553 (-0.925, -0.181) | 0.004 | 96.9% |  | 4 | 0.719 (0.657, 0.788) | 1.55×10^-12^ | 0.0% |  | 10 | 0.961 (0.923, 1.001) | 0.054 | 80.9% |  |
| America | 13 | -0.086 (-0.215, 0.052) | 0.221 | 80.5% |  | 8 | 0.565 (0.454, 0.702) | 2.61×10^-07^ | 37.9% |  | 5 | 0.983 (0.944, 1.024) | 0.407 | 36.5% |  |
| **Gender** |  |  |  |  | 0.644 |  |  |  |  | 0.777 |  |  |  |  | 0.460 |
| Female | 15 | -0.234 (-0.54, 0.072) | 0.134 | 96.5% |  | 7 | 0.714 (0.619, 0.824) | 3.84×10^-06^ | 41.4% |  | 5 | 0.899 (0.796, 1.016) | 0.252 | 65.4% |  |
| Male | 11 | -0.341 (-0.612, -0.071) | 0.013 | 94.9% |  | 5 | 0.543 (0.349, 0.844) | 0.007 | 69.2% |  | 3 | 0.825 (0.731, 0.932) | 0.012 | 0.0% |  |
| **Quality of included studies** | | |  |  | 0.044 |  |  |  |  | 0.757 |  |  |  |  | 0.639 |
| High | 26 | -0.105 (-0.144, -0.067) | 6.41×10^-08^ | 85.8% |  | 21 | 0.623 (0.539, 0.720) | 1.73×10^-10^ | 85.8% |  | 18 | 0.985 (0.973, 0.997) | 0.012 | 64.7% |  |
| Moderate | 50 | -0.331 (-0.425, -0.238) | 4.09×10^-12^ | 95.3% |  | 9 | 0.671 (0.543, 0.828) | 2.06×10^-04^ | 49.0% |  | 9 | 0.956 (0.920, 0.994) | 0.025 | 84.1% |  |

**^a^** Number of datasets. ***^b^*** The *P* value for the factor in meta-regression. LDL: low density lipoprotein cholesterol, HDL: high density lipoprotein cholesterol.
