# Supplementary material for: Blood lipid metabolism and the risk of gallstone disease: a multi-center study and meta-analysis
Source: Lipids Health Dis. 2022 Mar 2;21:26. doi: 10.1186/s12944-022-01635-9 (PMC8889751; doi:10.1186/s12944-022-01635-9)
Supplement: Supplementary file 10 — Additional file 10. Sensitivity analysis for the final meta-analyses. TC: total cholesterol, LDL-C: low-density lipoprotein cholesterol, HDL-C: high-density lipoprotein cholesterol, SD: standard deviation, OR: odd ratios. [file 12944_2022_1635_MOESM10_ESM.docx]

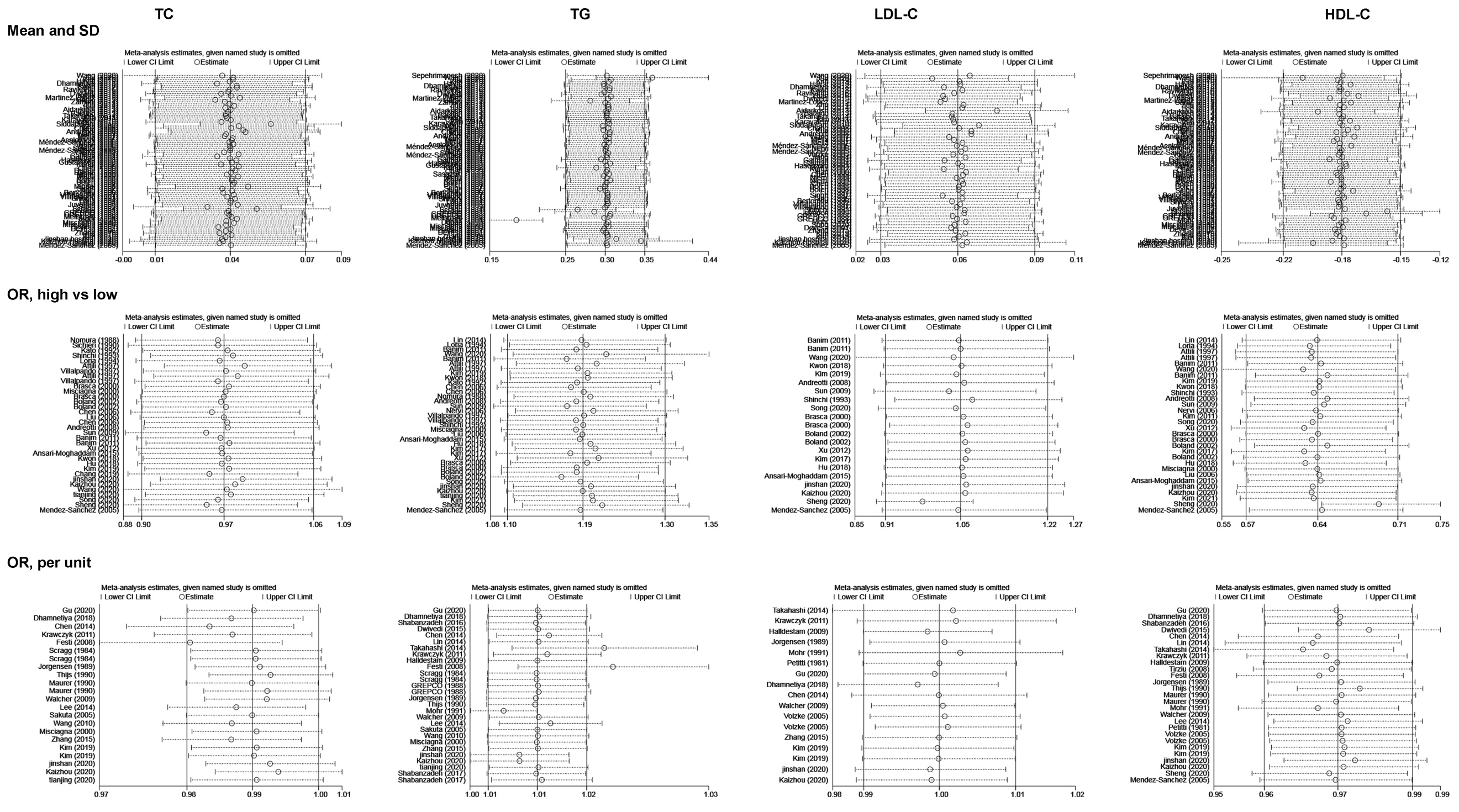


**Additional file 10.** Sensitivity analysis for the final meta-analyses. TC: total cholesterol, LDL-C: low-density lipoprotein cholesterol, HDL-C: high-density lipoprotein cholesterol, SD: standard deviation, OR: odd ratios
