# Supplementary material for: Blood lipid metabolism and the risk of gallstone disease: a multi-center study and meta-analysis
Source: Lipids Health Dis. 2022 Mar 2;21:26. doi: 10.1186/s12944-022-01635-9 (PMC8889751; doi:10.1186/s12944-022-01635-9)
Supplement: Supplementary file 11 — Additional file 11. Estimated regression parameters and standard errors in the dose-response meta-analysis. [file 12944_2022_1635_MOESM11_ESM.docx]

**Additional file 11.** Estimated regression parameters and standard errors in the dose-response meta-analysis.

|  | **Total cholesterol** | | **Triglycerides** | | **LDL-C** | | **HDL-C** | |
| --- | --- | --- | --- | --- | --- | --- | --- | --- |
|  | **β (se)** | ***P*** | **β (se)** | ***P*** | **β (se)** | ***P*** | **β (se)** | ***P*** |
| **Doses1** | 0.0117 (0.002) | <0.0001 | 0.0743 (0.019) | 0.001 | 0.0279 (0.006) | <0.0001 | -0.0871 (0.0265) | 0.004 |
| **Doses2** | -0.0125 (0.004) | 0.005 | -0.0889 (0.022) | <0.0001 | -0.0159 (0.006) | 0.022 | -0.0195 (0.0195) | 0.329 |
| ***P* for nonlinearity** | 0.0003 |  | 0.0005 |  | 0.0021 |  | 0.1562 |  |

se: standard error.
