# Supplementary material for: Blood lipid metabolism and the risk of gallstone disease: a multi-center study and meta-analysis
Source: Lipids Health Dis. 2022 Mar 2;21:26. doi: 10.1186/s12944-022-01635-9 (PMC8889751; doi:10.1186/s12944-022-01635-9)
Supplement: Supplementary file 12 — Additional file 12. Dose-response relationships between GSD risk and levels of (A) total cholesterol; (B) LDL-C; (C) Triglycerides; (D) HDL-C. [file 12944_2022_1635_MOESM12_ESM.docx]

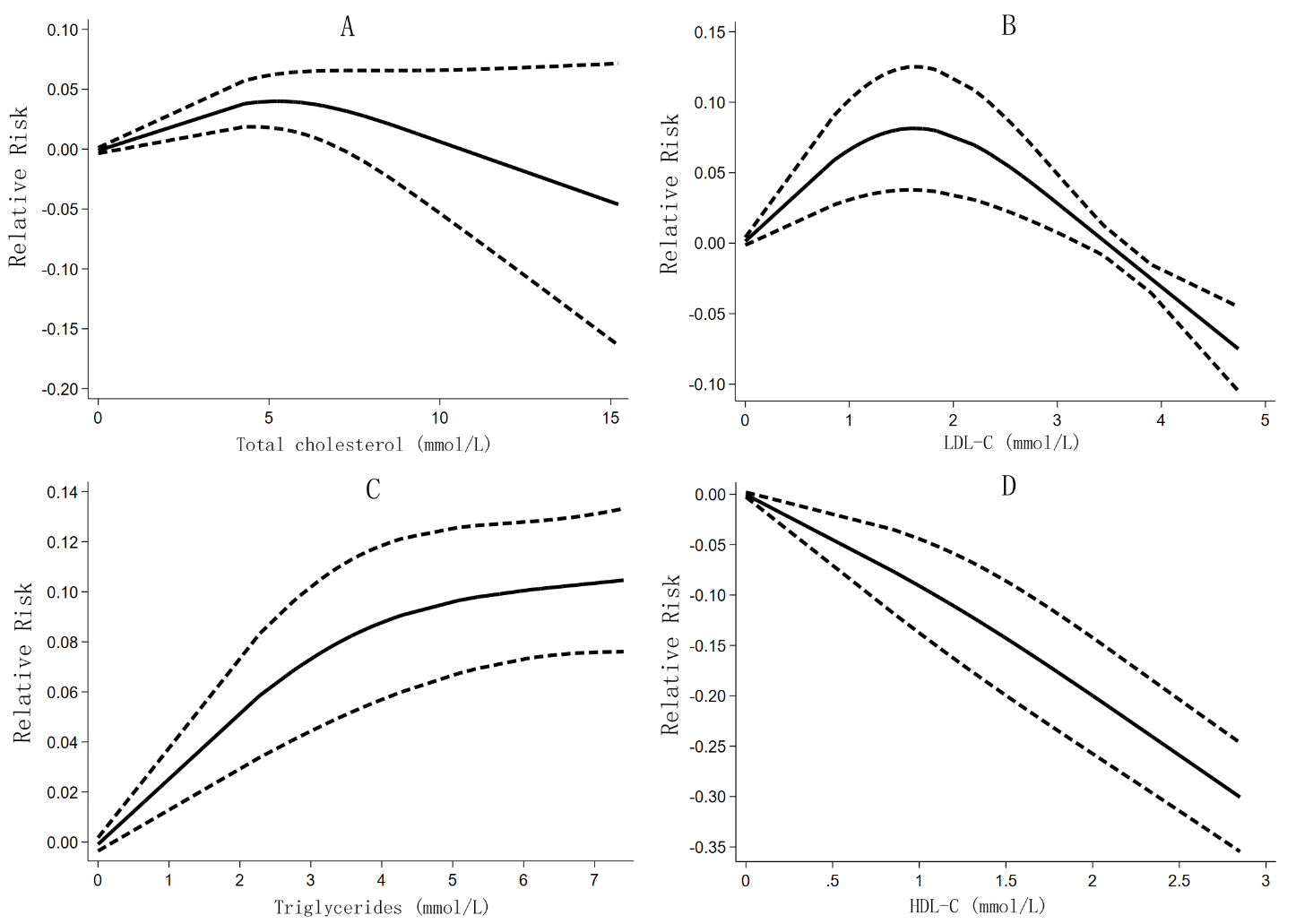


**Additional file 12.** Dose-response relationships between GSD risk and levels of (A) total cholesterol; (B) LDL-C; (C) Triglycerides; (D) HDL-C.
